# Cafeteria Diet Abstinence Induces Depressive Behavior and Disrupts Endocannabinoid Signaling in Dopaminergic Areas: A Preclinical Study

**DOI:** 10.2174/1570159X23666241107160840

**Published:** 2024-11-22

**Authors:** Marialuisa de Ceglia, Adele Romano, Maria Vittoria Micioni Di Bonaventura, Ana Gavito, Luca Botticelli, Emanuela Micioni Di Bonaventura, Marzia Friuli, Carlo Cifani, Fernando Rodríguez de Fonseca, Silvana Gaetani

**Affiliations:** 1 UGC de Salud Mental y Unidad Clínica de Neurología, Grupo de Neuropsicofarmacología, Instituto de Investigación Biomédica de Málaga (IBIMA), Universidad de Málaga-Hospital Universitario Regional de Málaga, 29010 Málaga, Spain;; 2 Department of Physiology and Pharmacology “V. Erspamer”, Sapienza University of Rome, Rome, Italy;; 3 School of Pharmacy, Pharmacology Unit, University of Camerino, Camerino, Italy

**Keywords:** Food addiction, abstinence, cafeteria diet, depression, endocannabinoid system, fatty acid amide hydrolase, dopamine

## Abstract

**Background:**

Alterations of dopamine (DA) transmission in the brain reward system can be associated with an addictive-like state defined as food addiction (FA), common in obese individuals. Subjects affected by FA experience negative feelings when abstinent from their preferred diet and may develop mood disorders, including depression, sustained by alterations in brain DA pathways.

**Objective:**

This study aims to investigate the impact of long-term abstinence from a palatable diet on depressive-like behavior in rats, exploring neurochemical alterations in monoamine and endocannabinoid signaling in DA-enriched brain regions, including ventral tegmental area, dorsolateral striatum, substantia nigra and medial prefrontal cortex.

**Methods:**

Rats underwent exposure and subsequent abstinence from a palatable cafeteria diet. During abstinence, animals were treated with fatty acid amide hydrolase (FAAH) inhibitor PF-3845 (10 mg/kg, intraperitoneal administration every other day). Lastly, animals were subjected to a forced swimming test, and their brains were dissected and processed for high-performance liquid chromatography measurement of monoamines and western blot analyses of markers of the endocannabinoid machinery.

**Results:**

After the withdrawal from the palatable diet, animals showed depressive-like behavior, coupled with significant variations in the concentration of brain monoamines and in the expression of endocannabinoid signalling machinery proteins in cited brain areas. Treatment with PF-3845 exerted an antidepressant-like effect and restored part of the alterations in monoaminergic and endocannabinoid systems.

**Conclusion:**

Overall, our results suggest that abstinence from a cafeteria diet provokes emotional disturbances linked to neuroadaptive changes in monoamines and endocannabinoid signalling in brain areas partaking to DA transmission that could partially be restored by the enhancement of endocannabinoid signalling through FAAH inhibition.

## 1 INTRODUCTION

Obesity is a growing disease in our society. According to the World Obesity Atlas, in 2020, 2.6 billion people were overweight, reaching 38% of the global population [1]. The obesity pandemic is largely attributed to overeating, and research efforts are dedicated to understanding the psychological bases of obesogenic eating habits. Interestingly, a great amount of evidence suggests that obesity is not only characterized by metabolic impairments but also by alterations of the “motivated behavior” that drives eating [2]. A variety of studies show that dopamine (DA) plays a crucial role in motivated behavior since it influences both perceived reward value and reward seeking. Alterations of DA transmission in the brain reward system [3, 4] might be involved in the establishment of an addicted-like state related to the dysregulation of eating behavior, defined as food addiction (FA) [5-8]. Moreover, restrained eating behavior has been associated with hypoactivity of DA neurons [7, 8], and interestingly, overeating may compensate for such alterations by promoting DA release, enhancing reward, and improving mood tone [9].

Individuals affected by FA experience negative feelings when abstinent from their preferred diet: several clinical studies have demonstrated a link between FA and depression [10-16]. Depression is a clinical condition characterized by emotional, neurovegetative, and neurocognitive symptoms. Patients experience anhedonia, feelings of worthlessness and guilt, suicidal ideation, and alterations in sleep and appetite [17]. A variety of mechanisms have been underlined for depression physiopathology; between them, the monoaminergic hypothesis associates depression with alterations of monoamine signalling in the brain [18]. In particular, depressed patients presented a reduction in brain serotonin (5HT) tone [19] and abnormal function of 5HT receptors [20], a reduction in brain noradrenaline (NA), and changes in adrenergic α_2_ autoreceptor activity [21]. Furthermore, variations in DA activity in the mesolimbic system regulating reward, hedonic, and motivated behaviors [22] have been proved.

DA is a catecholaminergic neurotransmitter, together with adrenaline and NA [23], and is mainly synthesized in the ventral tegmental area (VTA) and substantia nigra (SN). Projections from VTA to the prefrontal cortex (PFC), medial prefrontal cortex (mPFC), and nucleus accumbens (ACC) constitute the mesocorticolimbic system, which has a prominent role in reward and motivation, appetite-motivated behaviors, and regulation of hedonic aspects of food intake. Dysfunctions in the mesocorticolimbic system have been related to drug addiction, reward impairments, and mood disorders. On the other hand, neurons with soma in the SN and projecting to the dorsolateral striatum (DLS) form the nigrostriatal pathway and are principally involved in controlling movement, motivated behaviors, habit generation, and central pain modulation [24]. Interestingly, a substantial set of afferent inputs into the DLS originated in brain areas processing affective responses.

Behaviors such as eating involve decision-making and, consequently, the activation of the DA system. Interestingly, the endocannabinoid system (ECS) is a major regulator of DA activity and decision-making through its actions on inhibitory and excitatory synapses [2, 25] in the nigrostriatal and mesocorticolimbic pathway (for detailed information, see [26-28]). For this reason, ECS has been investigated as a target for obesity, addiction, and depression [29-31]. ECS consists of lipid transmitters collectively called endocannabinoids, namely anandamide (AEA) and 2-arachidonoyl-glycerol (2-AG), the enzymes for their synthesis (respectively N-acyl phosphatidylethanolamine-specific phospholipase D or NAPE-PLD and diacylglycerol lipase alpha/beta or DAGLα/β) and degradation (respectively Fatty-acid amide hydrolase or FAAH and Monoacylglycerol lipase or MAGL) and the cannabinoid receptors CB1 and CB2 [32].

Recently, we demonstrated that abstinence from a highly palatable diet could promote anxiety-like behavior [33] by inducing permanent changes in the expression of ECS in non-dopaminergic brain areas and that the treatment with the selective FAAH inhibitor PF-3845 [34] reversed such effects. Selective FAAH inhibitors are used to increase the levels of AEA and other acylethanolamides in the body; their administration results in modulation of the ECS and potential therapeutic effects. Up to date, FAAH inhibitors have shown potential application in the treatment of chronic pain, anxiety, and inflammation [35]. In the present study, we expanded our previous observations by focusing on the depressive aspect related to hedonic food consumption by investigating whether long-term abstinence from palatable food could induce depressive-like behavior in rats and alterations in monoamines and ECS signaling at the brain level. At the same time, we assessed whether treatment with the selective FAAH inhibitor PF-3845 exerted an antidepressant-like effect and restored the dysregulated monoaminergic and ECS.

## 2 MATERIALS AND METHODS

Animal samples analyzed in this study were originally collected for a previously published study [33], focused on anxiety-like behavior associated with food withdrawal, where a regional analysis of the ECS machinery in brain areas directly related to anxiogenic response was performed. The current investigation aims to build upon these findings by focusing on depressive-like behavior associated with food withdrawal and existing variations in the monoaminergic and ECS in brain areas related to reward processing.

### 2.1 Experimental Model

Adult male Wistar rats (Charles River, Italy) were group-housed under a 12 h light/dark cycle at constant temperature (20-22°C) and humidity (45-55%) with *ad libitum* access to water and standard chow (4RF18, Mucedola, Settimo Milanese, Italy) during 2 weeks before the beginning of the experiment.

On day 1, rats were randomly assigned to two different experimental groups:

- CHOW-fed group, with ad libitum access to water and standard chow.
- CAF-fed group, with ad libitum access to water, standard chow, and cafeteria diet.

As described in our previous studies [33, 36, 37], the cafeteria diet consisted of a mixture of different high-caloric palatable foods such as lard, muffins, cookies, chips, and cheese, individually weighed before being available to rats. Detailed diet content is shown in supplementary material section S1.1 and in [38]. This feeding regimen was followed from day 1 until day 40, until the end of phase 1 of the experiment, as shown in Fig. (**S1**). During phase 2 (days 41-68), the CAF-fed group underwent 28 days of abstinence from the cafeteria diet: animals could no longer access the cafeteria diet and were fed with ad libitum chow only. At the same time, during phase 2, all the rats were treated every other day either with the FAAH inhibitor PF-3845 (10 mg/kg; i.p.) or with vehicle injections (ethanol/tween 80/ saline in a proportion 5/5/90 v/v/v). All the experiments were performed following the European directive 2010/63/UE governing animal welfare and with the Italian Ministry of Health guidelines for the care and use of laboratory animals.

### 2.2 Body Weight and Food Intake Measurements

Results of body weight and food intake measurements were already published in [33].

### 2.3 Forced Swimming Test

On the last experiment day (day 68), 24 hours after the last administration of PF-3845, animals were subjected to a forced swimming test (FST). The test was carried out according to previous experiments [39, 40]. Further details are available in the supplementary material section S1.3.

### 2.4 Sacrifice and Brain Dissection

The day after FST, animals were euthanized by CO_2_ overdose. Brains were extracted, immediately snap frozen in 2-methyl butane, and stored at -80°C until further analyses. Using a cryostat (model HM550; Thermo Fisher Scientific, Kalamazoo, MI, USA), brains were microdissected into different regions of interest: the PFC, mPFC, dorsolateral striatum (DLS), ACC, amygdala (AMY), hypothalamus (HYPO), ventral pallidum (VPL), dorsal hippocampus (dHIPPO), ventral hippocampus (vHIPPO), periaqueductal grey (PAG), dorsal raphe (DR), locus coeruleus (LC), VTA, SN, lateral parabrachial nucleus (LPB). Details of dissection are shown in supplementary material section S1.4. Sections from both hemispheres were collected separately in microtubes, weighed to a high degree of accuracy, and stored at -80°C until processed.

### 2.5 HPLC Analyses of Monoamines and their Main Metabolites

All the sections from the right hemisphere were used for the HPLC analysis. Samples were prepared and run as described by [41, 42]. Further description of the HPLC technique is available in supplementary material section S1.5.

### 2.6 Western Blot Analysis

Sections from the left hemisphere were analyzed using a western blot. Total proteins from 5-15 mg of samples were extracted using 500 μL ice-cold cell lysis buffer as described in [43]. Western blot analysis was performed according to [44]. A detailed description of the Western blot analysis technique is available in supplementary material section S1.6.

### 2.7 Statistical Analysis

All data are expressed as mean ± SEM. Data from behavioral tests, HPLC, and western blot analysis were analyzed by two-way ANOVA (factors: diet and treatment). Subsequent multiple comparisons between groups were carried out by using Tukey post-hoc. Two-tail Bravais-Pearson correlation tests were performed for each experimental group to correlate different parameters. Statistical significance was set at *p* < 0.05. The software used for statistics and graphics were IBM SPSS Statistics 22 and GraphPad Prism 8.

## 3 RESULTS

Only significant variations among the different groups will be discussed to avoid extensive results description.

### 3.1 Forced Swimming Test

The results obtained by the FST showed a significant increase in immobility time in abstinent rats, when compared to CHOW VEH controls. Interestingly, PF-3845 administration in abstinent rats significantly reduced immobility time compared to vehicle administration, thus exerting an antidepressant-like effect. Two-way ANOVA for immobility time evidenced a significant effect for treatment (*p* < 0.05; F=6.879) and interaction (*p* < 0.01; F=10.701). It is important to underline that by the time of FST being performed, no significant difference was shown in the body weight, food intake, or locomotor activity among experimental groups [33], thus meaning that the difference in immobility time is not influenced by floating or poor aerobic activity.

These results are consistent with the ones obtained in our previous work in which we explored the possible beneficial effects of the pharmacological administration of PF-3845 on anxiety-like behaviour [33]. Indeed, diet abstinence evokes changes in both anxiety- and depression-related behaviors, which are significantly ameliorated by the administration of FAAH inhibitor PF-3845. Interestingly, significant correlations were shown among immobility time in FST and zone entries in OF (r^2^=0.3458; *p* < 0.001; negative correlation) and time spent in open arms in EPM (r^2^=0.2970; *p <* 0.05; negative correlation). Correlation graphs are included in supplementary material S2.2 (Figs. **S8** and **S9**).

Fig. (**1**) shows the results of FST along with the results of Tukey’s post-hoc.

### 3.2 HPLC Analyses of Monoamines and their Main Metabolites

Considering the existing link between brain monoamines and depression [45], we analyzed the concentrations of monoamines and their main metabolites in several brain areas.

#### 3.2.1 Dopaminergic Transmission

HPLC analyses revealed that withdrawal from the consumption of cafeteria diet provoked significant variations in the concentrations of DA: in particular, CAF VEH animals showed a significant decrease in DA concentration in DR and a significant increase in mPFC when compared to the CHOW VEH group. Importantly, PF-3845 administration dampened DA concentration in mPFC of cafeteria-abstinent rats, restoring it to control values (CHOW VEH).

Differently, abstinent animals showed a significant increase of DA main metabolite 3,4-Dihydroxyphenylacetic acid (DOPAC) in dHIPPO and a decrease of homovanillic acid (HVA) in ACC when compared to CHOW VEH. PF-3845 administration did not restore these variations but was associated with a significant increase in HVA concentrations in AMY and LC when compared to CAF VEH rats. Values expressed as MEAN ± SEM of the concentrations of DA, DOPAC, and HVA in ng/mg of wet tissue are shown below in Table **1**, along with the results of the Tukey post-hoc test.

#### 3.2.2 Serotoninergic Transmission

No significant changes in the concentration of 5HT were detected in abstinent rats compared to control ones, possibly due to the low number of samples and high interindividual variability. Abstinent animals treated with PF-3845 displayed a significant increase in 5HT concentration in SN when compared to both CAF VEH and CHOW PF animals. Differently, concentrations of 5-hydroxyindolacetic acid (5HIAA) were significantly lower in mPFC and VPL of abstinent rats when compared to the CHOW VEH group, suggesting a decrease in serotoninergic transmission in these areas. Values expressed as MEAN ± SEM of the concentrations of 5HT and 5HIAA in ng/mg of wet tissue are shown below in Table **2**, along with the results of Tukey post-hoc.

#### 3.2.3 Noradrenergic Transmission

Cafeteria diet abstinence caused a significant decrease in NA concentration in VPL of abstinent rats when compared to CHOW VEH ones. PF-3845 administration in abstinent rats partially restored NA concentrations in VPL and significantly increased NA content in the AMY when compared to CAF VEH. Values expressed as MEAN ± SEM of the concentrations of NA in ng/mg of wet tissue are shown below in Table **3**, along with the results of Tukey post-hoc. Unfortunately, we could not detect the NA main metabolite.

Supplementary material section S2.1 extensively describes two-way ANOVA parameters for HPLC analysis (p, F).

### 3.3 Pearson Correlations of Monoamine Levels

To assess whether inter-individual differences in monoamine contents within the various brain areas could provide information about their functional relationships [46, 47], we performed Pearson correlation analyses of the concentration of monoamines among the different brain areas in each experimental group.

#### 3.3.1 Dopaminergic Transmission

The patterns displaying Pearson correlations of DA across the different brain areas varied according to the experimental group: CHOW VEH animals presented only 5 correlations out of 120, differently from CAF VEH animals, which showed a significantly higher number (21 out of 120); suggesting that the abstinence from palatable diet impacted DA functional relationships among the areas analyzed, particularly in PFC, DLS, AMY, HYPO and vHIPPO, DR, SN, LPB. Interestingly, the administration of PF-3845 to abstinent rats (CAF PF group) reduced the correlation number (9 out of 120) and reestablished a more superimposable pattern to control animals. Correlative analysis is shown in Fig. (**2A**); only significant correlations were shown. The correlative analysis of DOPAC and HVA is reported in Supplementary Material section S2.2.

#### 3.3.2 Serotonergic Transmission

The patterns displaying Pearson correlations of 5HT across the different brain areas varied according to the experimental group: CHOW VEH animals presented 16 correlations out of 120, differently from CAF VEH animals, which showed a lower number (11 out of 120), suggesting that abstinence from palatable food decreased 5HT functional relationship among the different brain areas considered. Interestingly, the administration of PF-3845 in abstinent rats (CAF PF group) increased the number of correlations (27 out of 120) and reestablished a pattern of correlation superimposable to control animals. Correlative analysis is shown in Fig. (**2B**); only significant correlations were shown. Supplementary material section S2.2 includes the correlative analysis of 5HIAA.

No relevant variations were detected in Pearson correlations of NA, which are shown in supplementary material section S2.2.

### 3.4 Correlations between Behavioral Parameters and Monoamine/Metabolite Concentration in the Different Brain Areas

After evidencing that both the abstinence from the cafeteria diet and PF-3845 treatment can impact monoamine/metabolite concentration in several brain areas and change functional relationships through variation of correlation patterns, the next aim of our study was to establish whether immobility time in FST directly correlated with the concentration of monoamines/metabolites in selected brain areas.

Table **4** shows the significant correlations obtained among monoamine or their metabolites concentration and immobility time during FST. The results suggested that dopaminergic transmission was the most influenced: in fact, immobility time correlated to DA and its metabolites concentrations in PFC, mPFC, AMY, and DR. Differently, no significant correlations were observed for 5HT, whereas immobility time correlated with 5HIAA in ACC and AMY. Immobility time correlated with NA concentration only in ACC.

Table **S9** shows the significant correlations among monoamine/metabolite concentrations and parameters of anxiety obtained in our study [33].

### 3.5 Western Blot Analyses of the ECS in Dopaminergic Areas

In prior research [33], we linked anxiety in diet-abstinent rats to ECS variations in anxiety-related brain areas. Building on this, we explored ECS's role in dopaminergic transmission, expanding our analysis to key ECS proteins in DA-related brain regions [27].

**Fig. (3):**
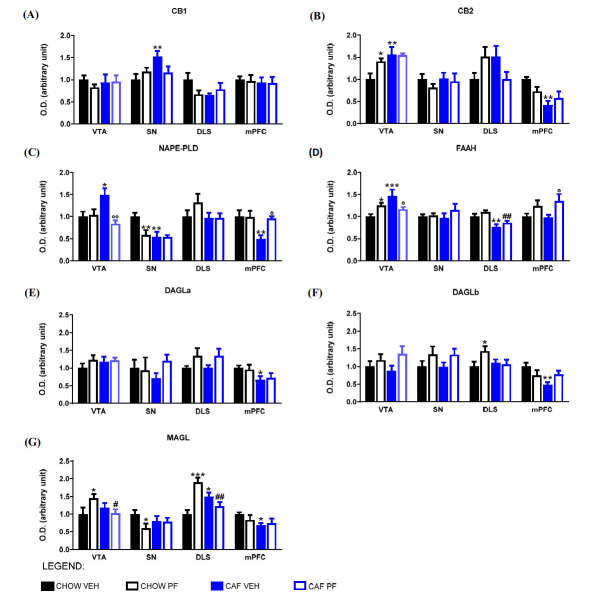
Protein expression of CB1 (**A**), CB2 (**B**), NAPE-PLD (**C**), FAAH (**D**), DAGLa (**E**), DAGLb (**F**), MAGL (**G**) in VTA, SN, DLS, mPFC measured by Western blot analysis and normalized to γ-adaptin. Values are expressed as MEAN ± SEM. Two-way ANOVA statistical analysis was performed, and Tukey post hoc results are shown in the figure. *p < 0.05; **p < 0.01 and ***p < 0.001 *vs.* CHOW VEH; °*p <* 0.05 and °°*p <* 0.01 *vs*. CAF VEH; #*p <* 0.05 and ##*p <* 0.01 *vs.* CHOW PF.

Our analysis revealed that the expression of cannabinoid receptors was altered by cafeteria diet abstinence in a site-specific manner (Figs. **3A** and **3B**): CB1 expression increased in the SN, while CB2 expression significantly increased in VTA and decreased in mPFC in CAF VEH rats when compared to CHOW VEH group. These changes were not reverted by PF-3845 treatment.

Other significant variations were noticed in the enzymes of synthesis and degradation of acylethanolamides, namely NAPE-PLD and FAAH (Figs. **3C** and **3D**). NAPE-PLD expression was significantly reduced in abstinent rats (CAF VEH) in both SN and mPFC, whereas it was significantly increased in the VTA.

PF-3845 administration to abstinent rats significantly prevented the variations in both mPFC and VTA. On the other hand, CAF VEH animals displayed a significant increase in FAAH expression in the VTA and a significant decrease in the DLS. PF-3845 administration to abstinent rats significantly dampened FAAH expression in the VTA and increased it in the mPFC.

Also, the expression of the enzymes of synthesis and degradation of 2-AG presented significant changes as a consequence of abstinence from the cafeteria diet (Figs. **3E**, **3F**, and **3G**). Abstinent animals displayed a significant decrease of DAGLa, DAGLb, and MAGL in mPFC when compared to CHOW VEH. Diet withdrawal also significantly increased MAGL expression in the DLS of rats when compared to CHOW VEH rats. In this case, PF-3845 treatment was not able to prevent such variations.

To sum up, the results obtained in this part of the study suggested that diet abstinence caused significant changes in the expression of the ECS in the areas of synthesis and projection of DA. In some cases, the PF-3845 treatment prevented such variations.

Fig. (**3**) shows the results of the western blot analysis and Tukey’s post-hoc results. Supplementary material section S2.3 extensively describes two-way ANOVA parameters (p, F). Supplementary material section 2.4 shows detailed images of western blot analysis, entire bands, and membranes.

### 3.6 Western Blot Analyses of FosB and ΔFosB in Dopaminergic Areas

FosB protein is a transcriptional factor involved in several molecular processes. Most importantly, its truncated form ΔFosB plays a central and crucial role in maintaining addiction, activating a series of transcription events that produce an addictive state. For this reason, we analyzed the expression of FosB and ΔFosB in reward areas to discriminate whether abstinence from palatable food consumption can produce the same molecular effects as drug abstinence.

We observed that diet abstinence provoked a significant decrease in FosB protein expression in the VTA, SN, and DLS, as compared to CHOW VEH. PF-3845 administration to abstinent rats produced a significant increase of FosB expression in ACC (as compared to both CAF VEH and CHOW PF animals) and in PFC (as compared to CHOW PF ones). Fig. (**4A**) shows the results of western blot analysis and Tukey’s post-hoc results.

ΔFosB followed a similar pattern of expression among the different experimental groups: abstinent animals presented a significant decrease in protein expression in SN, DLS, and PFC when compared to CHOW VEH. PF-3845 administration to abstinent rats significantly increased ΔFosB expression in PFC if compared to the CHOW PF group. Fig. (**4B**) shows the results of western blot analysis and Tukey’s post-hoc results.

Supplementary material section S2.3 extensively describes two-way ANOVA parameters (p, F). Supplementary material section 2.4 shows detailed images of western blot analysis, entire bands, and membranes.

### 3.7 Western Blot Analyses of the Expression of the ECS in DR

Since we observed significant variations in the pattern of correlation of concentrations of 5HT throughout the different brain areas, we also analyzed the expression of ECS in the area of synthesis of 5HT, the DR.

**Fig. (4):**
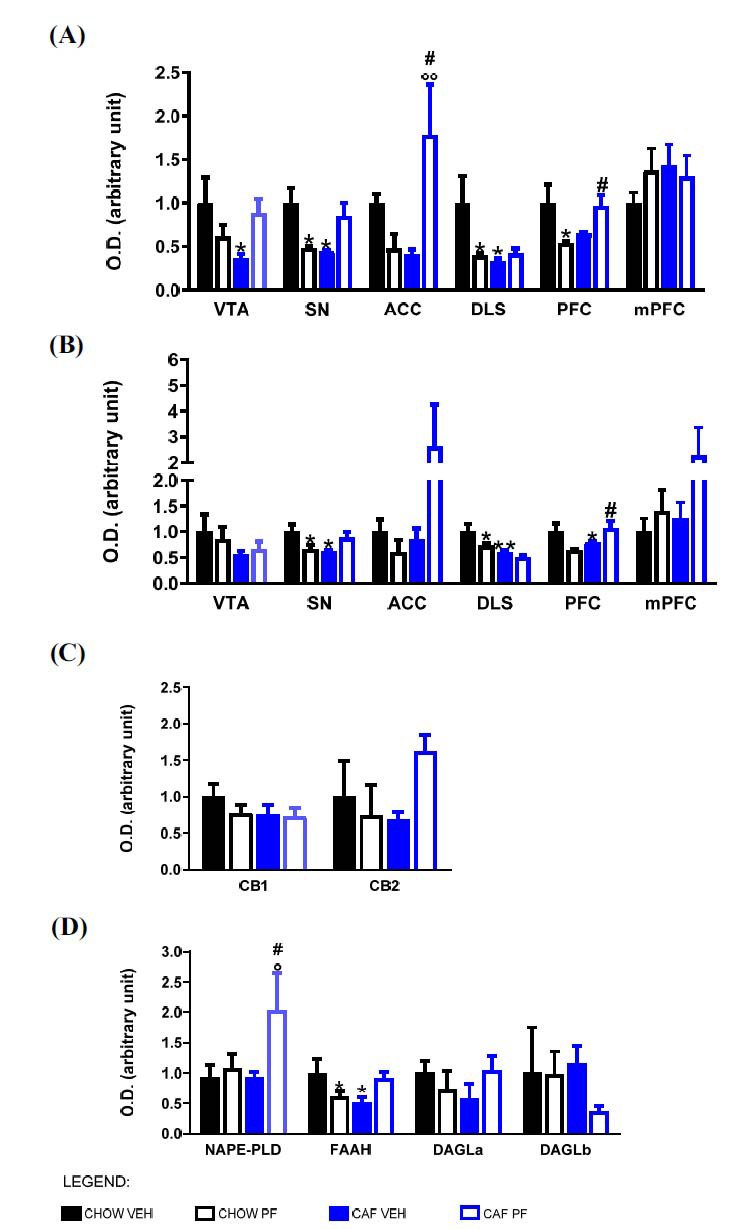
Protein expression of FosB (**A**), ΔFosB (**B**) in VTA, SN, DLS, and mPFC; protein expression of cannabinoid receptors (**C**) and enzymes of synthesis and degradation of cannabinoids (**D**) receptor in DR measured by Western blot analysis and normalized to γ-adaptin. Two-way ANOVA statistical analysis was performed, and Tukey post hoc results are shown in the figure. *p < 0.05 and **p < 0.01 *vs.* CHOW VEH; °*p <* 0.05 and °°*p <* 0.01 *vs.* CAF VEH; #*p <* 0.05 *vs.* CHOW PF. No proper staining was obtained for MAGL.

The expression of the cannabinoid receptors CB1 and CB2 did not show significant variations due to diet abstinence or treatment (Fig. **4C**). Differently, cafeteria diet abstinence caused a significant decrease in FAAH expression in this region. Moreover, PF-3845 treatment in abstinent rats significantly increased NAPE-PLD expression when compared to both CAF VEH and CHOW PF groups (Fig. **4D**).

Figs. (**4C** and **4D**) show the results of the western blot analysis and Tukey’s post-hoc results.

Supplementary material section S2.3 extensively describes two-way ANOVA parameters (p, F). Supplementary material section S2.4 shows detailed images of western blot analysis, entire bands, and membranes.

## 4 DISCUSSION

The results of the present study suggest that long-term abstinence from a palatable diet induced a depressive-like phenotype in rats, characterized by neurochemical alterations in monoamine and endocannabinoid signalling. In our previous study, already published and performed on the same set of animals [33], we demonstrated that the abstinence condition could induce a series of neuroadaptations leading to anxiety and that it could be alleviated through chronic treatment with PF-3845. Here, we expanded the beneficial effects of FAAH inhibitors by demonstrating that PF-3845 treatment was also able to prevent the depressive-like state associated with palatable food withdrawal.

Other preclinical studies already evidenced that cafeteria diet exposure provoked depressive-like behavior [48-50]: in addition to this observation, our results indicate that abstinence from palatable food consumption is not sufficient to reverse this mood alteration, suggesting the existence of neuroadaptive mechanisms that facilitate the persistence of the depressive response. The observation that depressive-like behavior shown by abstinent rats was coupled to variations in brain monoamine concentrations supports this hypothesis. In abstinent animals, we found decreased 5HIAA concentration in mPFC and decreased 5HT signaling in VPL, two neurochemical imbalances that were clinically associated with major depressive disorder [51, 52]. CAF VEH rats also presented a significant decrease in the concentration of DA in DR, in keeping with previous reports demonstrating that reduced DA concentration and decreased D2-like receptor activation in this area are associated with depressive-like phenotype [53, 54]. In addition, behavioral alterations observed in abstinent rats might also depend on the different patterns of correlations observed both in DA and 5HT, as revealed by Pearson analysis. These associations suggest that palatable food withdrawal changed the interactions among the different brain regions in both monoaminergic systems [46].

Some of these dysregulations could be reversed by administering FAAH inhibitor PF-3845: most importantly, PF-3845 treatment decreased immobility time in the FST, exerting an antidepressant-like effect possibly resulting from the stimulation of the ECS tone. These findings are consistent with previous studies demonstrating that PF-3845 and other FAAH inhibitors exhibit anti-depressant action in paradigms different from food withdrawal [54-57]. Abstinent rats treated with PF-3845 displayed increased DA concentration in DLS, which may also account for its antidepressant-like effect since deficient DA signaling in this area has been linked to depressive-like behavior [58, 59]. Similarly, PF-3845 increased DA and HVA in AMY: a large body of evidence suggests a role for AMY DA projections in depression [60, 61], even though further research is needed on this topic. Antidepressant-like action of PF-3845 in abstinent rats may also depend on increased NA signalling in AMY, as suggested by [62]. Moreover, the CAF PF group displayed a significant increase in FAAH in mPFC, which was previously associated with decreased expression of miRNAs coupled with depression [63]. Most importantly, PF-3845 administration in CAF PF rats provoked an increase in NAPE-PLD expression in the DR, resulting in increased AEA concentrations in this area and promotion of 5HT neuronal firing and basal synaptic transmission, as previously demonstrated [64, 65]. Consequently, PF-3845 chronic treatment in abstinent rats resulted in an increased number of correlations of 5HT in the CAF PF group, similar to what was reported upon the administration of other antidepressant drugs [66].

According to the present study, cafeteria diet abstinence appeared to have the greatest impact on DA transmission. The increased number of inter-area DA correlations in abstinent animals might be explained by the decreased FAAH activity in DR, which has been proven to facilitate DA transmission [67], and by variations in DA neuron excitability in SN and VTA. Such variations were sustained by significant alterations shown in CAF VEH rats in the expression of CB1 in the SN (which regulates synaptogenesis [68] and DA neuron firing [69, 70]) and of CB2 in the VTA (which regulates DA neuron excitability [71] and can be influenced by high-fat diet exposure [72]). Chronic administration of PF-3845 in abstinent rats reduced inter-area DA correlations, changing the interactions between homeostatic and reward processes [47]. Moreover, it increased 5HT concentration in SN, thus modulating DA release in the nigrostriatal pathway [73, 74]. PF-3845 treatment also restored the alterations in the expression of NAPE-PLD and FAAH observed in the VTA, contributing to the regulation of non-homeostatic feeding by tuning endocannabinoid signaling [75, 76]. ECS is an important modulator of DA signaling in reward areas: CB1 activation on GABAergic neurons in VTA disinhibits DA neurons and increases DA release in ACC, whereas CB1 activation on glutamatergic neurons in VTA suppresses glutamate release and finally decreases DA release in ACC. As a result, endocannabinoids have a significant impact on reward, motivation, mood regulation, and stress by regulating DA release [77]. Moreover, ECS also modulates the release of DA in the nigrostriatal pathway, affecting movement, addiction, and behaviour [78]. In addition, DA can also modulate ECS signaling in the brain [79].

Most importantly, several of the variations occurring in abstinent rats can be related to an addiction state, such as decreased 5HIAA in mPFC [80], increased DOPAC concentrations in dHIPPO [81, 82], reduction of FAAH in DLS [83], and increase in CB1 in SN [84]. Other changes were observed in the VTA of these animals, including alterations in the expression of CB2 and NAPE-PLD, involved in the modulation of rewarding and motivational effects of drugs of abuse [85-87], and in the synaptic plasticity associated with long-term alterations of addiction [88].

Even though these changes are comparable to those observed in drug addiction, other markers of addiction that we analyzed in abstinent rats suggest the existence of different molecular mechanisms controlling food withdrawal. FosB and ΔFosB are transcription factors associated with sensibilization and addiction to psychostimulant drugs and increase their expression in the addicted state through a mechanism mediated by the D1 receptor [89, 90]. In our case, withdrawal from a palatable diet caused an overall decrease of both FosB and ΔFosB in all the regions analyzed, which is in line with the study [91]. FAAH inhibitor administration to abstinent animals normalized the expression of NAPE-PLD in the VTA and increased the expression of FosB and ΔFosB in all regions except for DLS: this effect may depend on increased AEA tone, as demonstrated by [92].

Lastly, the CAF VEH group exhibited variations in the mPFC, which are compatible with exposure to aversive events and stress, such as a significant increase of tissular DA [93, 94] and an overall decrease in the expression of proteins partaking to the ECS [95, 96]. In this area, FAAH inhibition was effective in restoring DA concentrations and NAPE-PLD expression.

## CONCLUSION

To sum up, this work demonstrates that long-term abstinence from palatable food consumption provoked depressive-like behavior in rats. Behavioral variations were associated with changes in monoamine concentrations in different brain areas, DA being the most affected. Abstinence also caused significant modifications in the expression of proteins involved in ECS signaling in areas linked to DA transmission. Some of the changes observed both in monoamines and ECS were similar to those found in animal models of drug abuse, suggesting that FA shares pathways with addiction to drugs and results in long-term neuroadaptations. Administration of FAAH inhibitor PF-3845 to abstinent rats exerted an antidepressant-like effect and partially restored the variations observed in monoaminergic and endocannabinoid signalling. Combining this evidence with our previous work [33], it is possible to conclude that abstinence from palatable food consumption provokes anxiety and depressive-like behaviors and affects the brain’s ECS signalling. PF-3845 treatment significantly ameliorated behavioral patterns by restoring mood deficits through its antidepressant action. These pharmacological effects require the modulation of the ECS in DA transmission-relevant brain regions.

Ultimately, future studies are needed to assess whether female rats present similar alterations due to abstinence from the CAF diet. Also, additional experiments manipulating DA signaling while administering FAAH inhibitors may be needed to provide additional information about ECS effects on DA signaling/antidepressant-like behavior and clarify the mechanism through which these two systems interact. Moreover, additional experiments could be conducted to assess the course of depressive behavior, as well as to highlight the progression of alterations in monoaminergic and cannabinoid systems over time, planning a time course schedule during abstinence from palatable food consumption.

## Figures and Tables

**Fig. (1):**
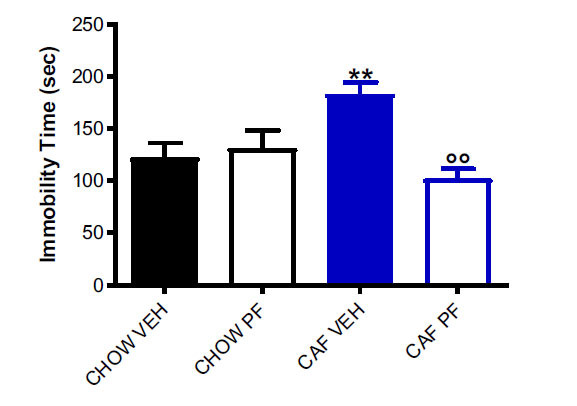
Results of forced swimming test. The graph shows the immobility time in seconds. Values are expressed as MEAN ± SEM. The picture shows the Tuckey post-hoc test for between-group comparisons: **p < 0.01 *vs.* CHOW VEH rats; °°*p <* 0.01 *vs*. CAF VEH.

**Fig. (2):**
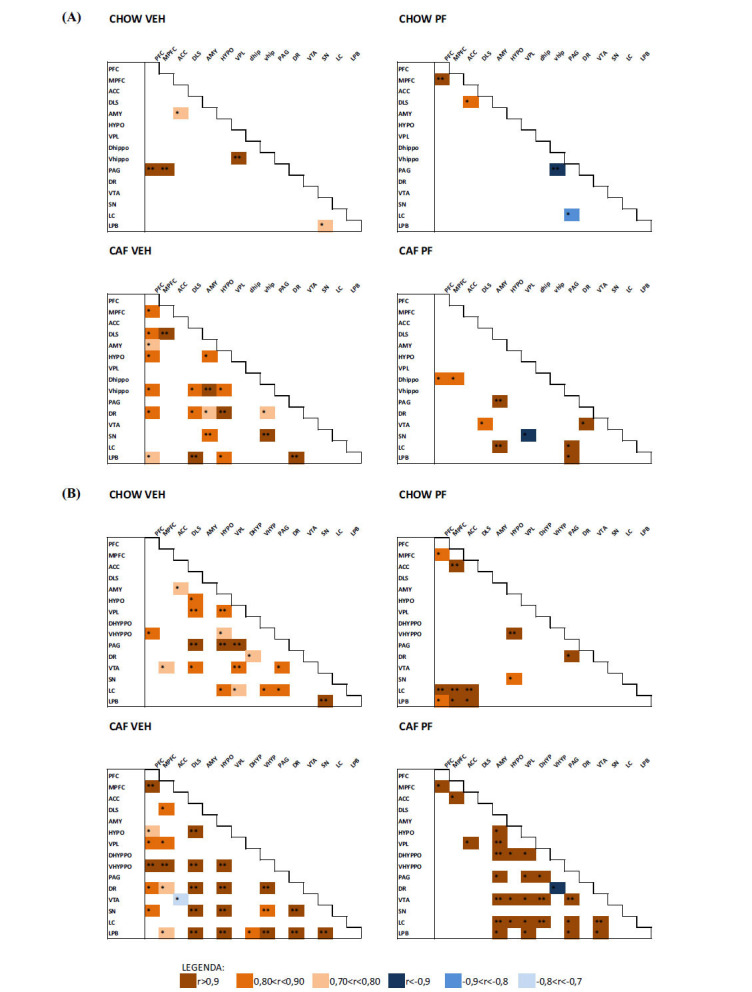
(**A**) Pearson’s correlative analysis of DA concentrations among the different brain areas in each experimental group. (**B**) Pearson’s correlative analysis of 5HT concentrations among the different brain areas in each experimental group. Only significant correlations are shown (*p < 0.05; **p < 0.01).

**Table 1:** Concentrations of DA, DOPAC, and HVA expressed as MEAN ± SEM in the different brain areas.

**DA (ng/mg Wet Tissue)**				
**Area**	**CHOW VEH**	**CHOW PF**	**CAF VEH**	**CAF PF**
VTA	0.9537 ± 0.1809	1.906 ± 0.3022 *	1.471 ± 0.3885	1.097 ± 0.3248
SN	0.1038 ± 0.0221	0.0936 ± 0.0425	0.109 ± 0.0490	0.1875 ± 0.0527
DR	0.08296 ± 0.03648	0.047 ± 0.01607	0.0145 ± 0.0037 *	0.0277 ± 0.0096
LC	0.1136 ± 0.0484	0.1919 ± 0.0914	0.1448 ± 0.0820	0.1526 ± 0.0675
AMY	1.28 ± 0.2722	0.789 ± 0.1991	0.4014 ± 0.1648	1.429 ± 0.6766
ACC	1.608 ± 0.5713	1.576 ± 0.5085	2.192 ± 0.856	1.494 ± 0.468
DLS	2.065 ± 0.6243	1.271 ± 0.2822	2.024 ± 0.4919	**3.079 ± 0.7833 #**
PFC	0.2759 ± 0.1578	0.3871 ± 0.1396	0.4171 ± 0.1868	0.22 ± 0.05679
mPFC	0.0267 ± 0.0156	0.0995 ± 0.0668	0.0964 ± 0.0498 *	**0.0227 ± 0.0063 °**
HIPO	0.1045 ± 0.0496	0.0425 ± 0.0147	0.0402 ± 0.0062	0.0712 ± 0.0165
VPL	0.2571 ± 0.1639	0.0572 ± 0.0215	0.1485 ± 0.0674	0.0744 ± 0.0247
dHIPPO	0.0063 ± 0.0036	0.0095 ± 0.0029	0.0163 ± 0.0106	0.0154 ± 0.0058
vHIPPO	0.2489 ± 0.0826	0.1133 ± 0.0253	0.1217 ± 0.0227	0.1164 ± 0.0285
PAG	0.0636 ± 0.0138	0.1341 ± 0.0154 ***	0.0394 ± 0.0073	**0.0209 ± 0.0037 ###**
LPB	0.01605 ± 0.0071	0.0104 ± 0.0028 *	0.0087 ± 0.0024	0.0089 ± 0.0017
**DOPAC (ng/mg Wet Tissue)**				
**Area**	**CHOW VEH**	**CHOW PF**	**CAF VEH**	**CAF PF**
VTA	0.1028 ± 0.04866	0.1473 ± 0.02814 *	0.1291 ± 0.03752	0.0899 ± 0.03797
SN	0.08728 ± 0.01342	0.084 ± 0.02334	0.0872 ± 0.03267	0.1006 ± 0.04369
DR	0.007882 ± 0.003522	0.003197 ± 0.0009358	0.003538 ± 0.001813	0.002586 ± 0.001004
LC	0.03112 ± 0.0111	0.03204 ± 0.01212	0.02265 ± 0.01086	0.01889 ± 0.01288
AMY	0.04629 ± 0.01733	0.05681 ± 0.02957	0.01944 ± 0.006382	0.02735 ± 0.00621
ACC	0.6553 ± 0.3671	0.4146 ± 0.1386	0.8405 ± 0.4834	0.3084 ± 0.1147
DLS	1.602 ± 0.4434	2.046 ± 0.3827	2.034 ± 0.6483	1.736 ± 0.2037
PFC	0.04754 ± 0.031	0.05847 ± 0.02132	0.09137 ± 0.03929	0.02824 ± 0.007709
mPFC	0.05842 ± 0.03051	0.08481 ± 0.02134	0.1363 ± 0.05438	0.04869 ± 0.01261
HIPO	0.1226 ± 0.01423	0.1364 ± 0.02348	0.08967 ± 0.01948	**0.06809 ± 0.005604 #**
VPL	0.1186 ± 0.03061	0.0534 ± 0.01632	0.1535 ± 0.08988	0.05214 ± 0.01077
dHIPPO	0.02909 ± 0.005253	0.03487 ± 0.003766	0.03116 ± 0.01505 *	0.02442 ± 0.004846
vHIPPO	0.01515 ± 0.005799	0.009263 ± 0.003917	0.006314 ± 0.002801	0.002992 ± 0.001929
PAG	0.003805 ± 0.001366	0.008408 ± 0.002413	0.005162 ± 0.002032	**0.006938 ± 0.005682 #**
LPB	0.01599 ± 0.006472	0.01227 ± 0.001394	0.008384 ± 0.001622	0.009554 ± 0.002703
**HVA (ng/mg Wet Tissue)**				
**Area**	**CHOW VEH**	**CHOW PF**	**CAF VEH**	**CAF PF**
VTA	0.06106 ± 0.01626	0.1128 ± 0.01754 *	0.09709 ± 0.02261	0.07517 ± 0.02792
SN	0.0437 ± 0.01025	0.05485 ± 0.0163	0.04801 ± 0.0145	0.08421 ± 0.023
DR	0.01346 ± 0.003353	0.0169 ± 0.01014	0.005174 ± 0.001996	0.01163 ± 0.006604
LC	0.01048 ± 0.003207	0.01225 ± 0.003388	0.03536 ± 0.008346	**0.07251 ± 0.0232 °#**
AMY	0.03089 ± 0.004611	0.03713 ± 0.009953	0.0126 ± 0.003427	**0.07884 ± 0.03856 °**
ACC	0.4541 ± 0.09401	0.3505 ± 0.07069	0.4154 ± 0.1595 *	0.2741 ± 0.07897
DLS	0.3933 ± 0.07654	0.4534 ± 0.05136	0.3931 ± 0.09595	0.4547 ± 0.04566
PFC	0.07101 ± 0.02322	0.08238 ± 0.02102	0.1051 ± 0.02494	0.06208 ± 0.01322
mPFC	0.02569 ± 0.01183	0.03683 ± 0.007626	0.04676 ± 0.01639	0.03352 ± 0.00746
HIPO	0.04129 ± 0.008424	0.05759 ± 0.01513	0.02664 ± 0.005632	0.03732 ± 0.005536
VPL	0.03985 ± 0.01577	0.01713 ± 0.003563	0.05078 ± 0.03185	0.0251 ± 0.005358
dHIPPO	0.01089 ± 0.001597	0.01399 ± 0.001865	0.01227 ± 0.004752	0.01476 ± 0.003613
vHIPPO	0.006374 ± 0.0009649	0.01009 ± 0.002661	0.004904 ± 0.0009783	**0.005173 ± 0.001223 #**
PAG	0.01017 ± 0.002213	0.02266 ± 0.008627	0.01433 ± 0.00279	0.05535 ± 0.04652
LPB	0.01648 ± 0.007669	0.0102 ± 0.003794	0.00932 ± 0.002382	0.01251 ± 0.005767

**Note:** *p < 0.05, ***p < 0.001 *vs.* CHOW VEH; °*p* < 0.05 *vs.* CAF VEH; #*p* < 0.05, ###*p* < 0.001 *vs.* CHOW PF.

**Table 2:** Concentrations of 5HT and 5HIAA expressed as MEAN ± SEM in the different brain areas.

**5HT (ng/mg Wet Tissue)**				
**Area**	**CHOW VEH**	**CHOW PF**	**CAF VEH**	**CAF PF**
VTA	0.2537 ± 0.08976	0.4828 ± 0.1328	0.3791 ± 0.1076	0.3258 ± 0.1366
SN	0.2343 ± 0.05734	0.1896 ± 0.04723	0.1376 ± 0.03744	**0.4099 ± 0.1048 # °°**
DR	0.2204 ± 0.1245	0.1542 ± 0.0943	0.03265 ± 0.009518	0.03043 ± 0.003889
LC	0.1988 ± 0.13	0.1409 ± 0.0776	0.09992 ± 0.04764	0.1294 ± 0.07448
AMY	0.08497 ± 0.04867	0.03835 ± 0.01714	0.02528 ± 0.007034	0.0622 ± 0.02711
ACC	0.0374 ± 0.0146	0.06298 ± 0.02686	0.0592 ± 0.02043	0.05422 ± 0.01626
DLS	0.0829 ± 0.02126	0.05603 ± 0.01249	0.05761 ± 0.01336	0.09937 ± 0.02835
PFC	0.2355 ± 0.0685	0.1508 ± 0.0461	0.1037 ± 0.02664	0.1142 ± 0.0298
mPFC	0.05789 ± 0.02178	0.06168 ± 0.03427	0.04678 ± 0.0136	0.04356 ± 0.006643
HIPO	0.2807 ± 0.07445	0.1533 ± 0.03978	0.1516 ± 0.03981	0.2444 ± 0.07998
VPL	0.2127 ± 0.06774	0.05227 ± 0.01043 *	0.06412 ± 0.03683	0.1408 ± 0.08382
dHIPPO	0.06865 ± 0.0226	0.06757 ± 0.01657	0.08319 ± 0.02912	0.1278 ± 0.05694
vHIPPO	0.2377 ± 0.08791	0.1011 ± 0.02022	0.1072 ± 0.02155	0.1165 ± 0.02861
PAG	0.1079 ± 0.07324	0.06795 ± 0.02353	0.03364 ± 0.01044	0.5217 ± 0.4888
LPB	0.07463 ± 0.03247	0.04155 ± 0.01389	0.07717 ± 0.02708	0.07261 ± 0.02855
**5HIAA (ng/mg Wet Tissue)**				
**Area**	**CHOW VEH**	**CHOW PF**	**CAF VEH**	**CAF PF**
VTA	0.7252 ± 0.302	1.394 ± 0.3504*	0.978 ± 0.2523	**0.4096 ± 0.134^#^**
SN	0.3735 ± 0.06073	0.3241 ± 0.03797	0.2694 ± 0.08284	0.3951 ± 0.1407
DR	0.362 ± 0.217	0.4371 ± 0.2261	0.06827 ± 0.02903	0.05711 ± 0.0203
LC	0.2696 ± 0.1784	0.1439 ± 0.05638	0.1598 ± 0.09637	0.1588 ± 0.08696
AMY	0.2729 ± 0.1171	0.138 ± 0.05454	0.07496 ± 0.02679	0.2848 ± 0.1182
ACC	0.4061 ± 0.2032	0.402 ± 0.09651	0.9082 ± 0.3908	0.4516 ± 0.1351
DLS	0.3178 ± 0.06591	0.3562 ± 0.02378	0.238 ± 0.08444	0.286 ± 0.03609
PFC	0.4109 ± 0.08206	0.6485 ± 0.04381	0.5713 ± 0.06777	0.4188 ± 0.1092
mPFC	0.2107 ± 0.07001	0.2837 ± 0.01718	0.1782 ± 0.04456**	0.2538 ± 0.03367
HIPO	0.1664 ± 0.02296	0.1879 ± 0.03641	0.1424 ± 0.04288	0.118 ± 0.02591
VPL	1.253 ± 0.2735	0.5915 ± 0.07821*	0.4311 ± 0.1617**	0.7651 ± 0.09265
dHIPPO	0.1815 ± 0.04966	0.1805 ± 0.02584	0.1325 ± 0.05753	0.1488 ± 0.0224
vHIPPO	0.6033 ± 0.1408	0.6477 ± 0.1133	0.6054 ± 0.07211	0.6369 ± 0.08144
PAG	0.07516 ± 0.02079	0.1774 ± 0.0973	0.05471 ± 0.02668	0.1819 ± 0.1381
LPB	0.08184 ± 0.02999	0.0441 ± 0.007913	0.04527 ± 0.01681	0.06371 ± 0.01752

**Note:** *p < 0.05, **p < 0.01 *vs.* CHOW VEH; °°*p <* 0.01 *vs.* CAF VEH; #*p <* 0.05 *vs.* CHOW PF.

**Table 3:** Concentrations of NA expressed as MEAN ± SEM in the different brain areas.

**NA (ng/mg Wet Tissue)**				
**Area**	**CHOW VEH**	**CHOW PF**	**CAF VEH**	**CAF PF**
VTA	0.6218 ± 0.2646	0.4807 ± 0.1061	0.3179 ± 0.09143	0.2205 ± 0.03512
SN	0.2994 ± 0.09451	0.2032 ± 0.0319	0.2294 ± 0.07559	0.4197 ± 0.1401
DR	0.2298 ± 0.1312	0.1546 ± 0.05018	0.05039 ± 0.0266	0.105 ± 0.05241
LC	0.2709 ± 0.1082	0.3767 ± 0.2128	0.2694 ± 0.154	0.1879 ± 0.09024
AMY	0.202 ± 0.05516	0.1338 ± 0.02259	0.07676 ± 0.03354	**0.2451 ± 0.07052 °**
ACC	0.1065 ± 0.02905	0.1365 ± 0.03633	0.229 ± 0.07834	0.1329 ± 0.05333
DLS	0.1206 ± 0.03317	0.1113 ± 0.01496	0.06675 ± 0.01818	0.07903 ± 0.01045
PFC	0.2281 ± 0.04308	0.2097 ± 0.03077	0.2199 ± 0.03385	0.1869 ± 0.03024
mPFC	0.1588 ± 0.05682	0.1786 ± 0.03663	0.1674 ± 0.02521	0.1659 ± 0.01284
HIPO	0.8467 ± 0.1116	0.6154 ± 0.09639	0.6828 ± 0.2043	0.839 ± 0.1793
VPL	0.3927 ± 0.08215	0.1556 ± 0.006305**	0.1155 ± 0.06234**	0.2288 ± 0.05057
dHIPPO	0.255 ± 0.06875	0.2666 ± 0.03766	0.3094 ± 0.1058	0.2783 ± 0.05633
vHIPPO	0.4649 ± 0.05507	0.3515 ± 0.06444**	0.3832 ± 0.04084	0.3383 ± 0.02491
PAG	0.1171 ± 0.03135	0.07755 ± 0.02751	0.145 ± 0.04739	0.04575 ± 0.02883
LPB	0.2278 ± 0.1293	0.1921 ± 0.07122	0.1635 ± 0.03892	0.2009 ± 0.08248

**Note:** **p < 0.01 *vs.* CHOW VEH; °*p* < 0.05 *vs.* CAF VEH.

**Table 4:** Significant correlations between monoamine/metabolite concentrations and immobility time during FST.

Dopaminergic transmission	**DA**				**DOPAC**				**HVA**			
	**AREA**	**r^2^**	***p***	**+/-**	**AREA**	**r^2^**	***p***	**+/-**	**AREA**	**r^2^**	***p***	**+/-**
	PFC	0.2239	*p <* 0.05	+	mPFC	0.2987	*p <* 0.05	+	mPFC	0.2084	*p <* 0.05	+
	mPFC	0.307	*p <* 0.001	+	ACC	0.2639	*p <* 0.05	+	ACC	0.197	*p <* 0.05	+
	AMY	0.817	*p <* 0.05	-					DR	0.2632	*p <* 0.05	-
	DR	0.2671	*p <* 0.05	-	-							
Serotonergic transmission	**5HIAA**											
	**AREA**	**r^2^**	***p***	**+/-**								
	ACC	0.3584	*p <* 0.01	+								
	AMY	0.2812	*p <* 0.05	-								
Noradrenergic transmission	**NA**											
	**AREA**	**r^2^**	***p***	**+/-**								
	ACC	0.2674	*p <* 0.05	+								

## Data Availability

Raw data were generated at the School of Pharmacy, Pharmacology Unit, University of Camerino (behavioral data); Department of Physiology and Pharmacology “V. Erspamer”, Sapienza University of Rome (HPLC data); UGC Salud Mental, Instituto de Investigación Biomédica de Málaga (IBIMA), Universidad de Málaga-Hospital Universitario Regional de Málaga (western blotting data). Data supporting the findings of this study are derived from the senior authors SG, FRF, and CC upon request.

## LIST OF ABBREVIATIONS

ACC: Nucleus Accumbens
AEA: Anandamide
AMY: Amygdala
CB1: Cannabinoid Receptors Type 1
CB2: Cannabinoid Receptors Type 2
DA: Dopamine
DAGLα: Diacylglycerol Lipase Alpha
DAGLβ: Diacylglycerol Lipase Beta
DLS: Dorsolateral Striatum
DOPAC: 3,4-Dihydroxyphenylacetic Acid
dHIPPO: Dorsal Hippocampus
DR: Dorsal Raphe
ECS: Endocannabinoid System
EPM: Elevated Plus Maze
FA: Food Addiction
FAAH: Fatty-acid Amide Hydrolase
FST: Forced Swimming Test
HPLC: High-performance Liquid Chromatography
HYPO: Hypothalamus
LC: Locus Coeruleus
LPB: Lateral Parabrachial Nucleus
MAGL: Monoacylglycerol Lipase
mPFC: Medial Prefrontal Cortex
NA: Noradrenaline
NAPE-PLD: N-Acyl Phosphatidylethanolamine-Specific Phospholipase D
OF: Open Field
PAG: Periaqueductal Grey
PFC: Prefrontal Cortex
SN: Substantia Nigra
VPL: Ventral Pallidum
vHIPPO: Ventral Hippocampus
VTA: Ventral Tegmental Area
5HIAA: 5-Hydroxyindoleacetic Acid
5HT: Serotonin
2-AG: 2-Arachidonoylglycerol
