# Supplementary material for: Cafeteria Diet Abstinence Induces Depressive Behavior and Disrupts Endocannabinoid Signaling in Dopaminergic Areas: A Preclinical Study
Source: Curr Neuropharmacol. 2024 Nov 22;23(4):458–74. doi: 10.2174/1570159X23666241107160840 (PMC12105268; doi:10.2174/1570159X23666241107160840)
Supplement: Supplementary file 1 [file CN-23-4-458_SD1.pdf]

Supplementary Material

Cafeteria Diet Abstinence Induces Depressive Behavior and Disrupts Endocannabinoid Signaling in Dopaminergic Areas: A Preclinical Study

Marialuisa de Ceglia[1,2,#], Adele Romano[2,#], Maria Vittoria Micioni Di Bonaventura[3], Ana Gavito[1], Luca Botticelli[3], Emanuela Micioni Di Bonaventura[3], Marzia Friuli[2], Carlo Cifani[3], Fernando Rodríguez de Fonseca[1,*] and Silvana Gaetani[2]

[1]*UGC de Salud Mental y Unidad Clínica de Neurología, Grupo de Neuropsicofarmacología, Instituto de Investigación Biomédica de Málaga (IBIMA), Universidad de Málaga-Hospital Universitario Regional de Málaga, 29010 Málaga, Spain;* [2]*Department of Physiology and Pharmacology "V. Erspamer", Sapienza University of Rome, Rome, Italy;* [3]*School of Pharmacy, Pharmacology Unit, University of Camerino, Camerino, Italy*

1875-6190/25 $65.00+.00 **© 2025 Bentham Science Publishers**

Supplementary Information Text

S1 Supplementary Materials and Methods

S1.1 Detailed diet content:

Table S1: The detailed content of the CHOW and CAFETERIA diet

Diet	Component	Energy content (kcal/g)	% protein content	% fat content	% carbohydrate content
CHOW	pellet	2.6	21	7	72
CAFETERIA	lard	9.0	0	100	0
CAFETERIA	sippets	5.5	5.8	55.5	37.7
CAFETERIA	cheese	4.2	35.4	64.6	0
CAFETERIA	Fonzies	5.3	5.0	54.9	38.9
CAFETERIA	mortadella	3.2	21.8	78.1	0
CAFETERIA	cookies	4.8	5.0	37.3	56.6
CAFETERIA	muffin	4.5	4.8	47.3	45.8

S1.2 Experimental paradigm:

Figure S1. Detailed experimental paradigm. Rats were exposed to a cafeteria-style diet (including chips, cheese, lard, muffin, cookies, etc.) for 40 days (CAF). A control group of rats with ad libitum access only to standard chow and water was also included in the study (CHOW). After the first 40 days of cafeteria diet exposure, rats underwent an abstinence period for 28 days, with no longer access to the cafeteria diet but still ad libitum access to standard chow. During abstinence, animals were treated either with the FAAH inhibitor PF-3845 (10 mg/kg, i.p.; PF) or their vehicle (VEH) administered every other day. Days of PF-3845 administration are indicated by red arrows.

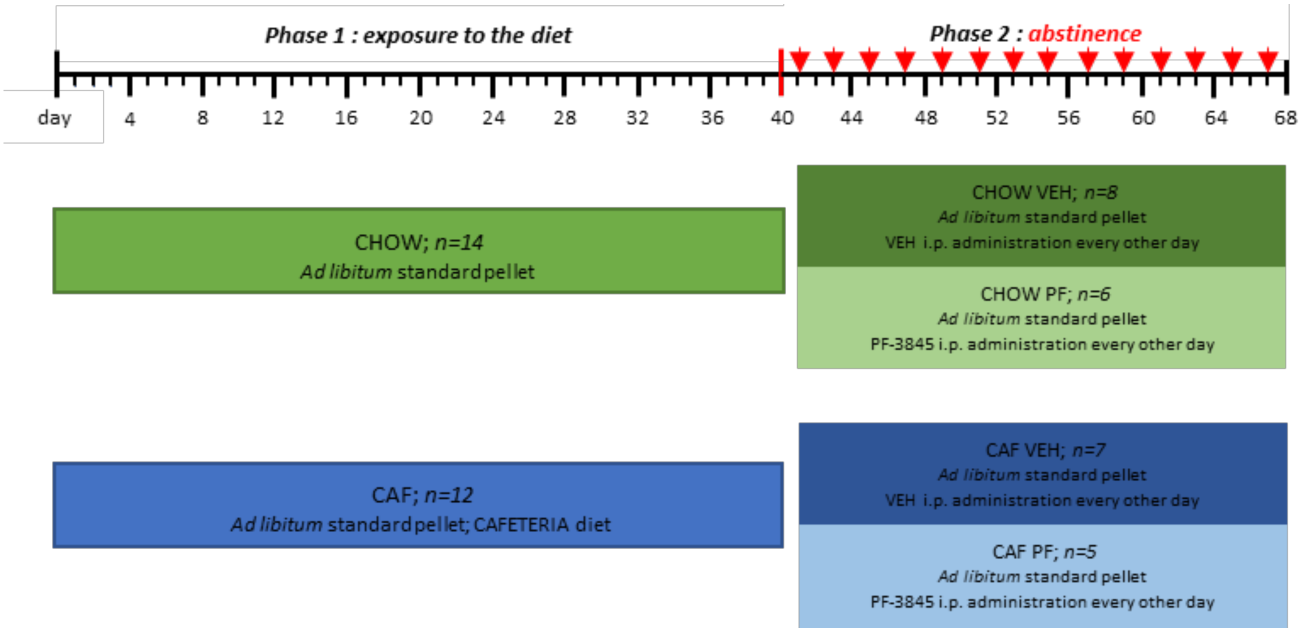

S1.3 Forced Swimming Test:

A forced Swimming Test (FST) was carried out in a sound-attenuated room illuminated by a dim red light (30 lux). Each rat was put in a plexiglass cylinder (diameter 29 cm; height 50 cm) filled with water at a temperature of 23-25 °C (Cifani et al., 2020; Vitale et al., 2017), to a depth that did not allow the rats rest at the bottom of the cylinder or escape. Each animal was subjected to two swimming sessions separated by 24 h: the first session (training, (Porsolt et al., 1977) lasted 15 min, whereas the second one, the only monitored, was 5 min long. Water was changed after every trial and animals were gently dried with a towel after swimming. During the second session, the parameter monitored was the immobility time, defined as floating with the absence of any movement except for those necessary for keeping the nose above water, indicative of depressive-like behavior (Yankelevitch-Yahav et al., 2015).

S1.4 Microdissection of regions of interest:

Brains were cut in 50 µm thick coronal sections, that were microdissected using a micro punch. Regions of interest were selected using Paxino's Brain Atlas, as shown below with representative images for each region (figure S2-S7).

Figure S2. Representative image of Figure 8 from Paxino's rat brain atlas. Dissected regions of interest in this area were mPFC (in orange) and PFC (in brown).

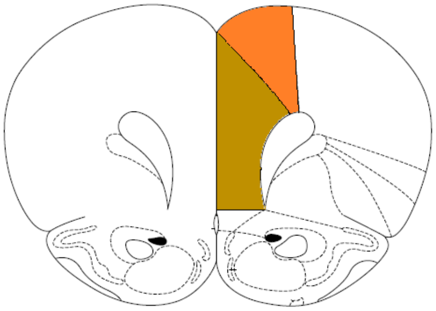

Figure S3. Representative image of Figure 11 from Paxino's rat brain atlas. Dissected regions of interest in this area were DLS (in red); ACC (in green); VPL (in light blue).

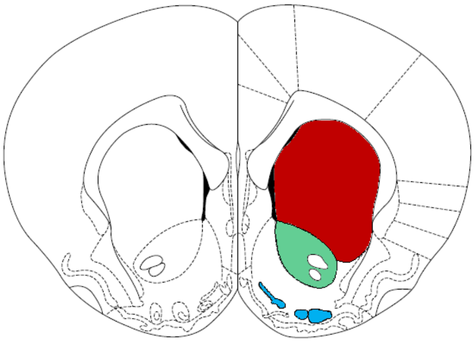

Figure S4. Representative image of Figure 35 from Paxino's rat brain atlas. Dissected regions of interest in this area were DHIP-PO (in violet); HYPO (in red); AMY (in blue).

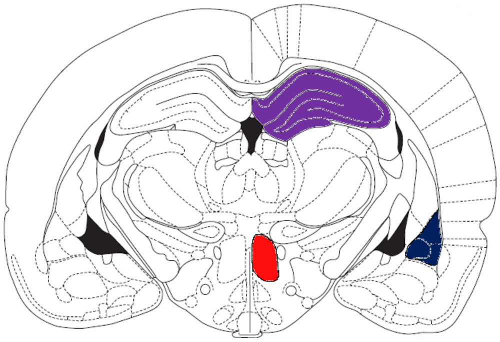

Figure S5. Representative image of Figure 44 from Paxino's rat brain atlas. Dissected regions of interest in this area were VHIP-PO (in green); SN (in brown); VTA (in grey).

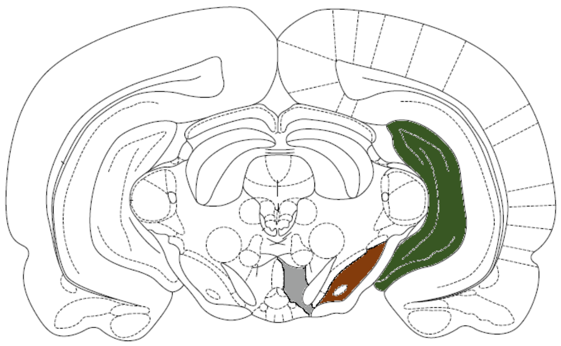

Figure S6. Representative image of Figure 51 from Paxino's rat brain atlas. Dissected regions of interest in this area were PAG (in pink); and DR (in brown).

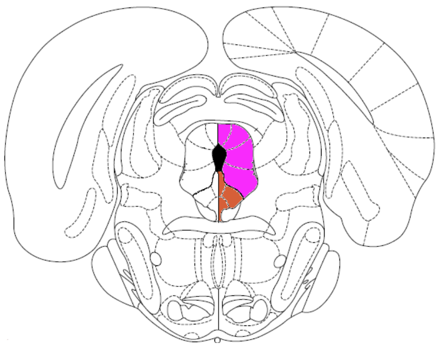

Figure S7. Representative image of Figure 58 from Paxino's rat brain atlas. Dissected regions of interest in this area were LC (in grey); and LPB (in green).

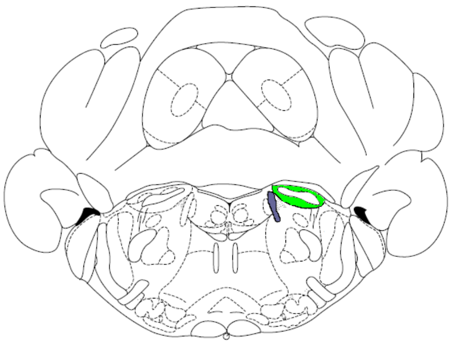

S1.5 HPLC analysis procedure:

Samples were ultrasonicated in ice-cold 0.1 M perchloric acid and then centrifuged at 15000g for 20 min at 4°C as described by Cassano et al. (Cassano et al., 2009). Supernatants were collected and used for monoamines and monoamine metabolites assay. Samples of the same region of all experimental groups were run in parallel to eliminate the possibility of run effects. Tissue levels of monoamines (dopamine or DA, noradrenaline or NA, serotonin or 5HT) and both 5HT and DA metabolites (5HIAA or 2-(5-Hydroxy-1H-indol-3-yl)acetic acid; DOPAC or 3,4-Dihydroxyphenylacetic acid; HVA or homovanillic acid) were detected and quantified. Monoamines and their metabolites were analyzed by microbore HPLC, the detection was accomplished with a Unijet cell (BAS) with a 6-mm diameter glassy carbon electrode set at +650 mV vs an Ag/AgCl reference electrode, connected to an electrochemical amperometric detector (INTRO, Antec Leyden, Netherlands). The analytes were separated using a SphereClone 150-mm × 2-mm column (3-µm packing) and a mobile phase composed of 85 mM of sodium acetate, 0.34 mM EDTA, 15 mM sodium chloride, 0.81 mM of octane sulphonic acid sodium salt, 6% methanol (v/v) (pH = 4.85) delivered at a flow rate of 800 µl/min for a total runtime of 35 min. For each analysis, a set of standards containing various concentrations of each compound (monoamines and their metabolites) was prepared in the acid solution to obtain appropriate calibration curves. The concentrations of neurotransmitters were determined by linear interpolation from standard curves; we normalized their concentration to the weight of the wet tissue sample.

S1.6 Western blot analysis procedure:

Sections from the left hemisphere were analyzed by western blot. Total proteins from 5–15 mg of samples were extracted using 500 µL ice-cold cell lysis buffer. A quantity of 50 µg of protein was resolved on a 4–12% (Bis-Tris) Criterion XT Precast Gel (Bio-Rad Laboratories, Inc., Hercules, CA, USA, cat. number: 3450124), and then transferred onto nitrocellulose membranes (Bio-Rad Laboratories, Inc., Hercules, CA, USA). Total protein content was visualized after staining with Ponceau red. Membranes were blocked in TBS-T (50 mM Tris-HCl (pH 7.6), 200 mM NaCl, and 0.1% Tween 20) with 2% albumin fraction V from BSA (Roche, Mannheim, Germany) for 1h at room temperature. For specific protein detection, the membrane was incubated overnight at 4 °C in TBS-T containing 2% BSA and the corresponding primary antibody (Supplementary material table S2 shows all the primary antibodies used in Western Blot experiments). γ-adaptin was used as a reference protein. After several washes in TBS-T containing 1% Tween 20, an HRP-conjugated anti-rabbit or anti-mouse IgG (H+L) secondary antibody (Promega, Madison, WI, USA) diluted 1:10000 was added, followed by incubation for 1 h at room temperature. After extensive washing in TBS-T, the membranes were incubated for 1 min with the Western Blotting Luminol Reagent kit (Bio-Rad Laboratories, Inc., Hercules, CA, USA), and the specific protein bands were visualized and quantified by chemiluminescence using a Chemi-Doc TM MP Imaging System (Bio-Rad, Barcelona, Spain). Quantification of the results was performed by using ImageJ software. The results are expressed as target protein/γ-adaptin ratios. Results for CHOW VEH animals' protein levels were arbitrarily set as 1.

Table S2: Primary antibodies used for protein expression by Western blotting

Antigen	Manufacturing and host	Datasheet link for Ab specificity	Dilution	Molecular weight
γ-adaptin	BDTransduction (610385), mouse	https://www.bdbiosciences.com/en-pl/products/reagents/microscopy-imaging-reagents/immunofluorescence-reagents/purified-mouse-anti-adaptin.610385	1/2000	100
CB1	Abcam (ab23703), rabbit	https://www.abcam.com/cannabinoid-receptor-i-antibody-ab23703.html	1/200	52
CB2	Abcam (ab3561), rabbit	https://www.abcam.com/cannabinoid-receptor-ii-antibody-ab3561.html	1/200	40
DAGLa	Biorbyt (156533), rabbit	https://www.biorbyt.com/dagla-antibody-cf594-orb156533-cf594.html	1/100	115
DAGLb	Biorbyt (182976), rabbit	https://www.biorbyt.com/daglb-antibody-orb182976.html	1/100	74
NAPE-PLD	Abcam (ab95397), rabbit	https://www.abcam.com/nape-pld-antibody-ab95397.html	1/1000	46
MAGL	Abcam (ab24701), rabbit	https://www.abcam.com/monoacylglycerol-lipasemgl-antibody-ab24701.html	1/500	33
FAAH	Cayman (101600), rabbit	https://www.caymanchem.com/product/101600	1/200	65
Fos B	Santa Cruz Biotechnology (102, sc-48), rabbit	https://datasheets.scbt.com/sc-48.pdf	1/200	36-31

Only the band corresponding to the exact molecular weight was quantified for each staining obtained. Quantification of delta-Fos B was done by quantifying the band at 31 kDa in the staining of Fos B.

S1.7 Statistical analysis procedure:

All data are expressed as mean ± SEM. Data from behavioral tests, HPLC, and western blot analysis were analyzed by two-way ANOVA (factors: diet and treatment). Subsequent multiple comparisons between groups were carried out by using Tukey post-hoc. Two-tail Bravais – Pearson correlation tests were performed for each experimental group to correlate different parameters. Statistical significance was set at p< 0.05. The software used for statistics and graphics were IBM SPSS Statistics 22 and GraphPad Prism 8

S2 Supplementary Results

S2.1 Statistical analysis of HPLC results:

Table S3: Results of two-way ANOVA statistical analysis for DA concentration in the different brain areas. The two factors considered were diet (CHOW, CAF) and treatment (VEH, PF). Significant results are in italics.

area	F diet		F treatment		F interaction		df
PFC	0,008	(p=0.929)	0,089	(p=0.769)	1,143	(p=0.300)	21
mPFC	1,373	(p=0.255)	1,931	(p=0.180)	2,471	(p=0.132)	24
ACC	0,157	(p=0.697)	0,330	(p=0.573)	0,275	(p=0.607)	20
DLS	3,226	(p=0.087)	0,076	(p=0.785)	3,085	(p=0.094)	25
AMY	0,102	(p=0.753)	0,516	(p=0.482)	4,136	(p=0.058)	21
HIPO	0,451	(p=0.511)	0,341	(p=0.567)	3,093	(p=0.097)	21
VPL	0,252	(p=0.622)	2,265	(p=0.152)	0,478	(p=0.499)	20
dHYPPO	1,617	(p=0.221)	0,036	(p=0.853)	0,107	(p=0.747)	21
vHYPPO	1,876	(p=0.189)	2,415	(p=0.139)	2,063	(p=0.169)	21
PAG	**33,128**	***(p<0.001)***	**4,734**	***(p<0.05)***	**13,887**	***(p<0.01)***	21
DR	**4,716**	***(p<0.05)***	0,669	(p=0.422)	1,374	(p=0.254)	26
VTA	0,219	(p=0.645)	0,859	(p=0.365)	**4,508**	***(p<0.05)***	25
SN	1,297	(p=0.271)	0,618	(p=0.443)	1,039	(p=0.322)	21
LC	0,003	(p=0.957)	0,337	(p=0.570)	0,225	(p=0.641)	20
LPB	1,129	(p=0.304)	0,436	(p=0.518)	0,496	(p=0.492)	20

Table S4: Results of two-way ANOVA statistical analysis for DOPAC concentration in the different brain areas. The two factors considered were diet (CHOW, CAF) and treatment (VEH, PF). Significant results are in italics.

	F diet		F treatment		F interaction		df
PFC	0,063	(p=0.805)	0,931	(p=0.348)	1,873	(p=0.189)	21
mPFC	0,408	(p=0.531)	0,878	(p=0.362)	3,046	(p=0.099)	21
ACC	0,015	(p=0.904)	1,444	(p=0.247)	0,205	(p=0.657)	20
DLS	0,020	(p=0.889)	0,029	(p=0.866)	0,760	(p=0.396)	20
AMY	2,079	(p=0.168)	0,223	(p=0.643)	0,004	(p=0.948)	21
HIPO	**7,010**	***(p<0.05)***	0,097	(p=0.759)	1,433	(p=0.246)	23
VPL	0,117	(p=0.737)	2,864	(p=0.110)	0,135	(p=0.718)	20
dHYPPO	**8,811**	***(p<0.01)***	0,612	(p=0.443)	0,036	(p=0.851)	24
vHYPPO	3,709	(p=0.071)	1,378	(p=0.257)	0,107	(p=0.748)	21
PAG	2,226	(p=0.152)	0,400	(p=0.535)	3,891	(p=0.063)	23
DR	1,511	(p=0.236)	1,956	(p=0.180)	0,858	(p=0.367)	21

VTA	0,000	(p=0.995)	1,403	(p=0.249)	**4,309**	***(p<0.05)***	25
SN	0,076	(p=0.786)	0,029	(p=0.867)	0,078	(p=0.784)	21
LC	0,845	(p=0.372)	0,015	(p=0.905)	0,040	(p=0.845)	20
LPB	1,982	(p=0.178)	0,121	(p=0.732)	0,445	(p=0.514)	20

Table S5: Results of two-way ANOVA statistical analysis for HVA concentration in the different brain areas. The two factors considered were diet (CHOW, CAF) and treatment (VEH, PF)—significant results are in italics.

	F diet		F treatment		F interaction		Df
PFC	0,344	(p=0.565)	0,166	(p=0.689)	1,209	(p=0.287)	21
mPFC	0,634	(p=0.437)	0,009	(p=0.926)	1,194	(p=0.290)	21
ACC	4,112	(p=0.058)	0,722	(p=0.407)	0,949	(p=0.343)	22
DLS	0,000	(p=0.994)	0,832	(p=0.375)	0,000	(p=0.991)	20
AMY	0,213	(p=0.649)	**5,722**	***(p<0.05)***	0,938	(p=0.344)	25
HIPO	2,836	(p=0.110)	1,692	(p=0.211)	0,073	(p=0.790)	21
VPL	0,269	(p=0.611)	1,766	(p=0.202)	0,007	(p=0.936)	20
dHYPPO	0,118	(p=0.735)	0,801	(p=0.383)	0,010	(p=0.923)	21
vHYPPO	3,671	(p=0.068)	1,410	(p=0.248)	1,752	(p=0.199)	26
PAG	0,582	(p=0.457)	1,227	(p=0.284)	0,349	(p=0.563)	20
DR	0,958	(p=0.341)	0,510	(p=0.485)	0,047	(p=0.831)	21
VTA	0,029	(p=0.867)	1,071	(p=0.312)	3,421	(p=0.078)	26
SN	1,011	(p=0.329)	2,001	(p=0.175)	0,560	(p=0.464)	21
LC	**10,763**	***(p<0.01)***	1,809	(p=0.193)	4,016	(p=0.058)	25
LPB	0,210	(p=0.653)	0,085	(p=0.775)	0,800	(p=0.384)	20

Table S6: Results of two-way ANOVA statistical analysis for 5HT concentration in the different brain areas. The two factors considered were diet (CHOW, CAF) and treatment (VEH, PF). Significant results are in italics.

	F diet		F treatment		F interaction		df
PFC	3,319	(p=0.086)	0,645	(p=0.433)	1,060	(p=0.318)	21
mPFC	0,384	(p=0.543)	0,000	(p=0.990)	0,022	(p=0.883)	21
ACC	0,089	(p=0.769)	0,222	(p=0.644)	0,489	(p=0.495)	20
DLS	0,200	(p=0.661)	0,136	(p=0.717)	2,888	(p=0.109)	20
AMY	0,396	(p=0.537)	0,029	(p=0.867)	2,153	(p=0.161)	21
HYPO	0,101	(p=0.755)	0,084	(p=0.776)	3,379	(p=0.084)	21
VPL	0,342	(p=0.567)	0,666	(p=0.427)	**5,338**	***(p<0.05)***	20
dHIPPO	1,238	(p=0.281)	0,420	(p=0.526)	0,463	(p=0.506)	21
vHIPPO	1,524	(p=0.234)	1,867	(p=0.190)	2,454	(p=0.136)	21
PAG	0,580	(p=0.457)	0,809	(p=0.382)	1,123	(p=0.305)	20
DR	3,717	(p=0.071)	0,179	(p=0.677)	0,157	(p=0.697)	21
VTA	0,017	(p=0.897)	0,529	(p=0.477)	1,365	(p=0.259)	21
SN	3,560	(p=0.074)	**8,821**	***(p<0.01)***	**4,676**	***(p<0.05)***	24
LC	0,396	(p=0.538)	0,026	(p=0.873)	0,249	(p=0.625)	20
LPB	0,404	(p=0.534)	0,507	(p=0.487)	0,291	(p=0.597)	20

Table S7: Results of two-way ANOVA statistical analysis for 5HIAA concentration in the different brain areas. The two factors considered were diet (CHOW, CAF) and treatment (VEH, PF). Significant results are in italics.

	F diet		F treatment		F interaction		df
PFC	0,204	(p=0.658)	0,308	(p=0.586)	**6,459**	***(p<0.05)***	21
mPFC	**7,252**	***(p<0.05)***	0,364	(p=0.554)	3,088	(p=0.095)	23
ACC	1,362	(p=0.260)	0,950	(p=0.344)	0,916	(p=0.353)	20
DLS	2,046	(p=0.172)	0,678	(p=0.422)	0,008	(p=0.928)	20
AMY	0,088	(p=0.770)	0,190	(p=0.669)	4,006	(p=0.062)	21
HYPO	1,950	(p=0.181)	0,002	(p=0.966)	0,464	(p=0.505)	21
VPL	1,747	(p=0.202)	0,017	(p=0.896)	**8,376**	***(p<0.01)***	23
dHIPPO	0,993	(p=0.333)	0,036	(p=0.852)	0,045	(p=0.834)	21
vHIPPO	1,805	(p=0.197)	2,000	(p=0.175)	2,573	(p=0.127)	21
PAG	0,007	(p=0.933)	1,506	(p=0.238)	0,018	(p=0.896)	20
DR	4,025	(p=0.061)	0,036	(p=0.851)	0,066	(p=0.800)	21
VTA	3,246	(p=0.085)	0,650	(p=0.429)	**5,004**	***(p<0.05)***	26
SN	0,037	(p=0.849)	0,199	(p=0.661)	1,045	(p=0.321)	21
LC	0,173	(p=0.683)	0,309	(p=0.586)	0,300	(p=0.591)	20
LPB	0,185	(p=0.673)	0,240	(p=0.631)	2,034	(p=0.173)	20

Table S8: Results of two-way ANOVA statistical analysis for NA concentration in the different brain areas. The two factors considered were diet (CHOW, CAF) and treatment (VEH, PF). Significant results are in italics.

	F diet		F treatment		F interaction		df
PFC	0,198	(p=0.662)	0,549	(p=0.469)	0,044	(p=0.836)	21
mPFC	0,003	(p=0.956)	0,061	(p=0.807)	0,084	(p=0.775)	21
ACC	1,297	(p=0.271)	0,402	(p=0.535)	1,463	(p=0.244)	20
DLS	4,164	(p=0.058)	0,005	(p=0.945)	0,262	(p=0.616)	20
AMY	0,171	(p=0.684)	0,998	(p=0.329)	**4,972**	***(p<0.05)***	26
HYPO	0,039	(p=0.845)	0,062	(p=0.806)	1,660	(p=0.215)	21
VPL	1,663	(p=0.211)	1,266	(p=0.273)	**6,861**	***(p<0.05)***	25
dHIPPO	0,228	(p=0.639)	0,020	(p=0.890)	0,095	(p=0.761)	21
vHIPPO	0,143	(p=0.710)	**6,648**	***(p<0.05)***	2,650	(p=0.118)	25
PAG	0,003	(p=0.957)	3,927	(p=0.065)	0,727	(p=0.406)	20
DR	2,378	(p=0.141)	0,019	(p=0.891)	0,765	(p=0.394)	21
VTA	3,655	(p=0.073)	0,654	(p=0.430)	0,022	(p=0.884)	21
SN	0,665	(p=0.426)	0,275	(p=0.607)	2,542	(p=0.129)	21
LC	0,407	(p=0.532)	0,007	(p=0.936)	0,395	(p=0.539)	20
LPB	0,103	(p=0.753)	0,000	(p=0.992)	0,177	(p=0.679)	20

S2.2 Additional Pearson Correlation Analysis:

Figure S8. Study of correlation among immobility time in FST and time spent in open arms in EPM.

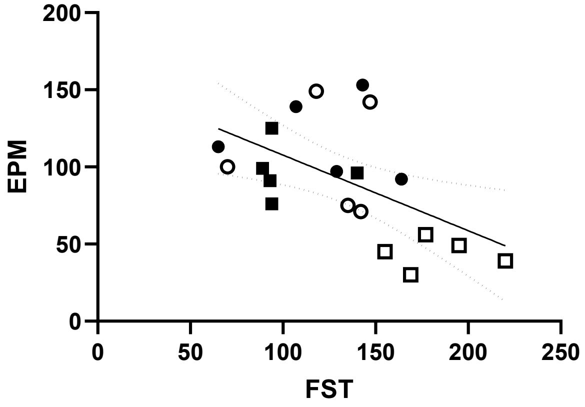

Goodness of Fit	
R squared	0.2970
Sy.x	32.08
Is slope significantly non-zero?	
F	7.604
DFn, DFd	1, 18
P value	0.0130

Figure S9. Study of correlation among immobility time in FST and zone entries in OF.

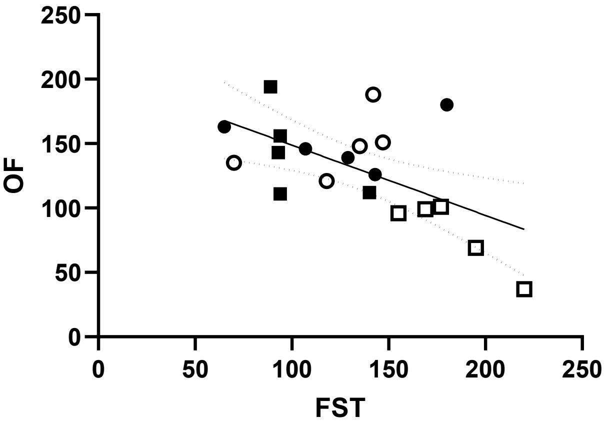

Goodness of Fit	
R squared	0.3458
Sy.x	32.53
Is slope significantly non-zero?	
F	9.514
DFn, DFd	1, 18
P value	0.0064

Table S9. Significant correlations between monoamine/metabolite concentrations and parameters of anxiety-like behavior

Significant correlations between monoamine/metabolite concentrations and time spent in open arms in EPM

	DA				DOPAC			
	AREA	r^2	p	+/-	AREA	r^2	p	+/-
**Dopaminergic transmission**	DR	0,2378	p<0.05	+	AMY	0,1972	p<0.05	+
	**5HIAA**							
	AREA	r^2	p	+/-				
**Serotonergic transmission**	AMY	0,239	p<0.05	+				
	VPL	0,2262	p<0.05	+				

Significant correlations between monoamine/metabolite concentrations and zone entries in OF

	DOPAC			
	AREA	r^2	p	+/-
**Dopaminergic transmission**	DLS	0,2345	p<0.05	-
	**5HIAA**			
	AREA	r^2	p	+/-
**Serotonergic transmission**	ACC	0,3392	p<0.05	-
	**NA**			
	AREA	r^2	p	+/-
**Noradrenergic transmission**	ACC	0,2826	p<0.05	-
	PAG	0,2007	p<0.05	-

Figure S10. Pearson's correlative analysis of DOPAC concentrations among the different brain areas in each experimental group. Only significant correlations are displayed (*p<0.05; **p<0.01).

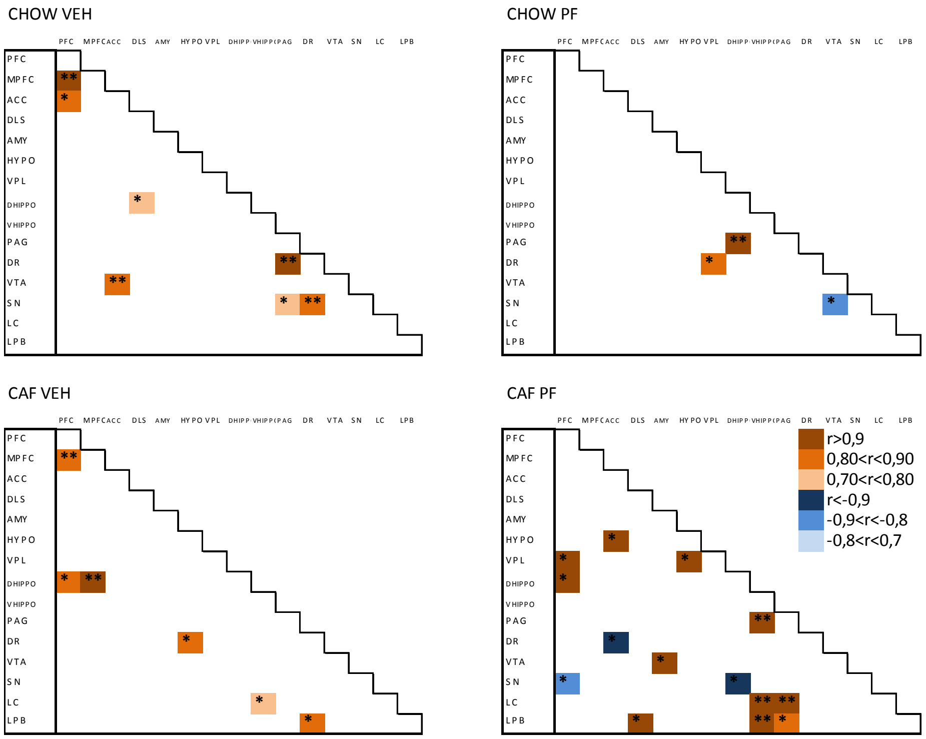

Figure S11. Pearson's correlative analysis of HVA concentrations among the different brain areas in each experimental group. Only significant correlations are displayed (*p<0.05; **p<0.01).

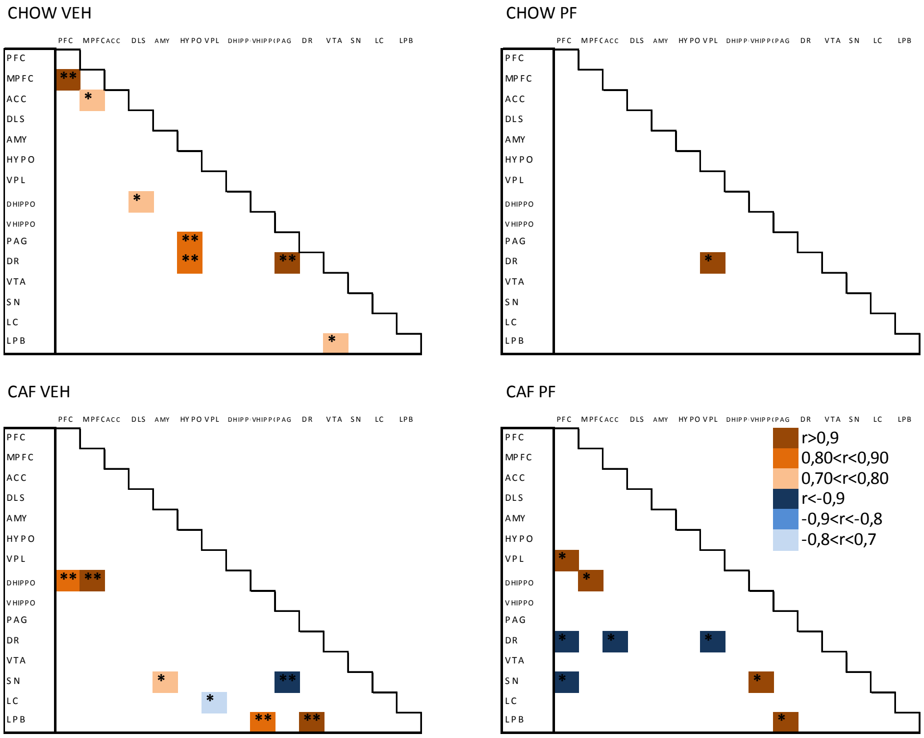

Figure S12. Pearson's correlative analysis of 5HIAA concentrations among the different brain areas in each experimental group. Only significant correlations are displayed (*p<0.05; **p<0.01).

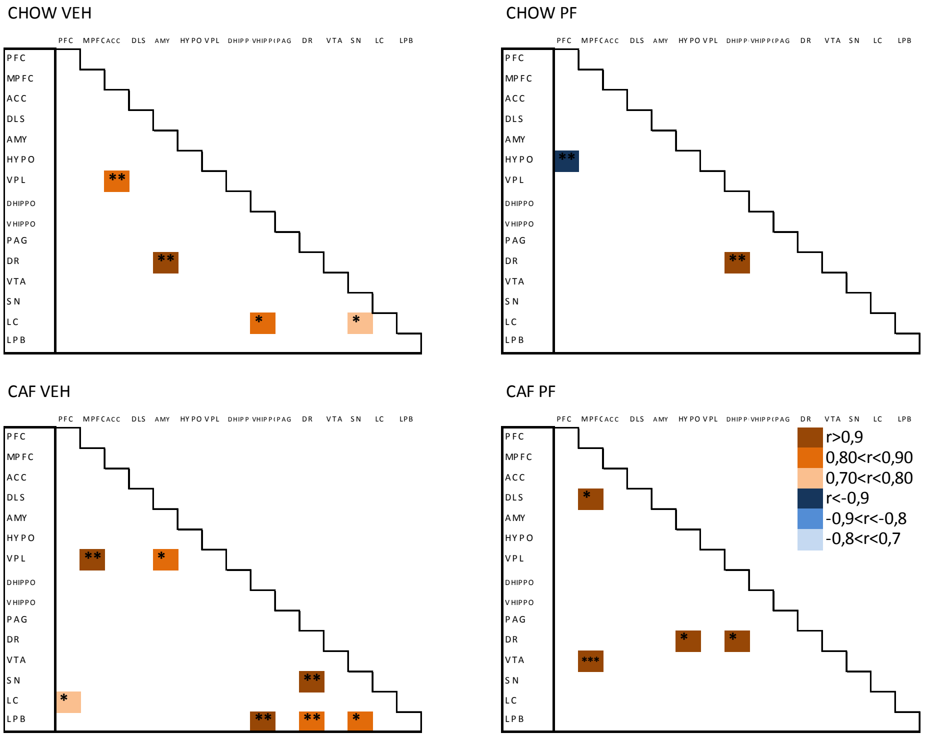

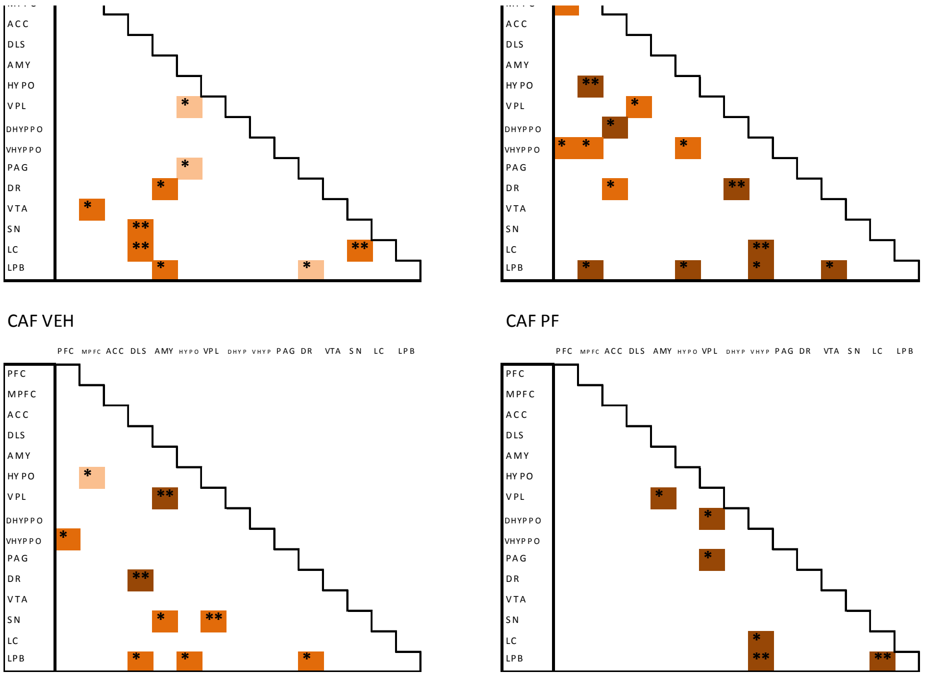

Figure S13. Pearson's correlative analysis of NA concentrations among the different brain areas in each experimental group. Only significant correlations are displayed (*p<0.05; **p<0.01).

S2.3 Statistical analysis of western blot results:

Table S10: Results of two-way ANOVA statistical analysis for protein expression obtained by western blot analysis in the different brain areas. The two factors considered were diet (CHOW, CAF) and treatment (VEH, PF). Significant results are in italics.

PROTEIN	AREA	F diet		F treatment		F interaction		df
	VTA	0,076	(p=0.785)	0,374	(p=0.547)	0,581	(p=0.454)	23
	SN	4,126	(p=0.054)	0,540	(p=0.470)	**4,760**	***(p<0.05)***	23
**CB1**	DLS	0,976	(p=0.333)	0,753	(p=0.394)	3,770	(p=0.064)	24
	mPFC	0,196	(p=0.662)	0,046	(p=0.832)	0,007	(p=0.934)	23
	DR	1,208	(p=0.283)	0,851	(p=0.365)	0,538	(p=0.470)	25
	VTA	**9,429**	***(p<0.01)***	2,945	(p=0.100)	3,271	(p=0.084)	23
	SN	0,318	(p=0.579)	0,870	(p=0.361)	0,222	(p=0.642)	23
**CB2**	DLS	0,000	(p=1,000)	0,000	(p=1,000)	**5,682**	***(p<0.05)***	24
	mPFC	**12,005**	***(p<0.01)***	0,310	(p=0.583)	4,171	(p=0.053)	23
	DR	0,206	(p=0.654)	0,589	(p=0.450)	3,642	(p=0.068)	25
	VTA	1,388	(p=0.253)	**6,378**	***(p<0.05)***	**7,855**	***(p<0.05)***	23
	SN	**7,165**	***(p<0.05)***	**4,955**	***(p<0.05)***	**4,936**	***(p<0.05)***	23
**NAPE-PLD**	DLS	0,001	(p=0.975)	0,367	(p=0.551)	2,610	(p=0.120)	24
	mPFC	**5,226**	***(p<0.05)***	3,655	(p=0.069)	3,926	(p=0.060)	23
	DR	0,206	(p=0.654)	0,589	(p=0.450)	3,642	(p=0.068)	25
	VTA	**4,953**	***(p<0.05)***	0,086	(p=0.772)	**10,663**	***(p<0.01)***	23
	SN	0,261	(p=0.615)	1,090	(p=0.307)	0,754	(p=0.395)	23
**FAAH**	DLS	**20,538**	***(p<0.001)***	3,355	(p=0.078)	0,001	(p=0.981)	24
	mPFC	0,134	(p=0.717)	**6,988**	***(p<0.05)***	0,377	(p=0.545)	23
	DR	0,928	(p=0.345)	0,034	(p=0.855)	**8,003**	***(p<0.01)***	25
	VTA	0,320	(p=0.577)	1,016	(p=0.324)	0,473	(p=0.499)	23
	SN	0,002	(p=0.965)	0,644	(p=0.431)	1,160	(p=0.293)	23
**DAGLa**	DLS	0,000	(p=0.992)	**4,625**	***(p<0.05)***	0,000	(p=0.992)	24
	mPFC	**6,489**	***(p<0.05)***	0,000	(p=1,000)	0,183	(p=672)	23
	DR	0,034	(p=0.855)	0,120	(p=0.732)	2,035	(p=0.167)	25
	VTA	0,018	(p=0.894)	3,471	(p=0.066)	0,733	(p=0.401)	23
	SN	0,004	(p=0.950)	**4,071**	***(p<0.05)***	0,002	(p=0.964)	23
**DAGLb**	DLS	1,072	(p=0.311)	2,152	(p=0.156)	3,148	(p=0.089)	24
	mPFC	**4,789**	***(p<0.05)***	0,029	(p=0.866)	**5,618**	***(p<0.05)***	23
	DR	0,213	(p=0.649)	0,685	(p=0.416)	0,594	(p=0.488)	25
	VTA	0,721	(p=0.405)	0,969	(p=0.337)	4,076	(p=0.055)	23
**MAGL**	SN	0,001	(p=0.975)	2,958	(p=0.099)	2,444	(p=0.132)	23
	DLS	0,536	(p=0.471)	**5,744**	***(p<0.05)***	**21,169**	***(p<0.001)***	24
	mPFC	3,749	(p=0.065)	0,316	(p=0.579)	1,117	(p=0.302)	23
	VTA	1,015	(p=0.334)	0,125	(p=0.730)	**6,091**	***(p<0.05)***	16
	SN	4,058	(p=0.067)	2,625	(p=0.131)	4,577	(p=0.054)	16
**FOSb**	ACC	1,519	(p=0.249)	2,212	(p=0.171)	**11,007**	***(p<0.01)***	13
	DLS	4,058	(p=0.057)	2,625	(p=0.131)	4,577	(p=0.054)	16
	PFC	0,074	(p=0.790)	0,332	(p=0.575)	**9,347**	***(p<0.05)***	16
	MPFC	0,622	(p=0.441)	0,291	(p=0.597)	1,213	(p=0.286)	21
	VTA	1,682	(p=0.224)	0,006	(p=0.938)	0,274	-0,612	14
	SN	0,679	(p=0.426)	0,102	(p=0.755)	**9,453**	***(p<0.05)***	16
**delta FOSb**	ACC	1,451	(p=0.259)	0,802	(p=0.394)	1,973	(p=0.194)	13
	DLS	**13,331**	***(p<0.01)***	4,377	(p=0.058)	1,253	(p=0.285)	16
	PFC	0,998	(p=0.337)	0,111	(p=0.745)	**9,009**	***(p<0.05)***	16
	MPFC	0,890	(p=0.359)	1,407	(p=0.252)	0,258	(p=0.618)	21

S2.4 Western blot detailed membranes images:

Figure S14. Ponceau Red and complete staining of bands used for Western blot analysis of VTA.

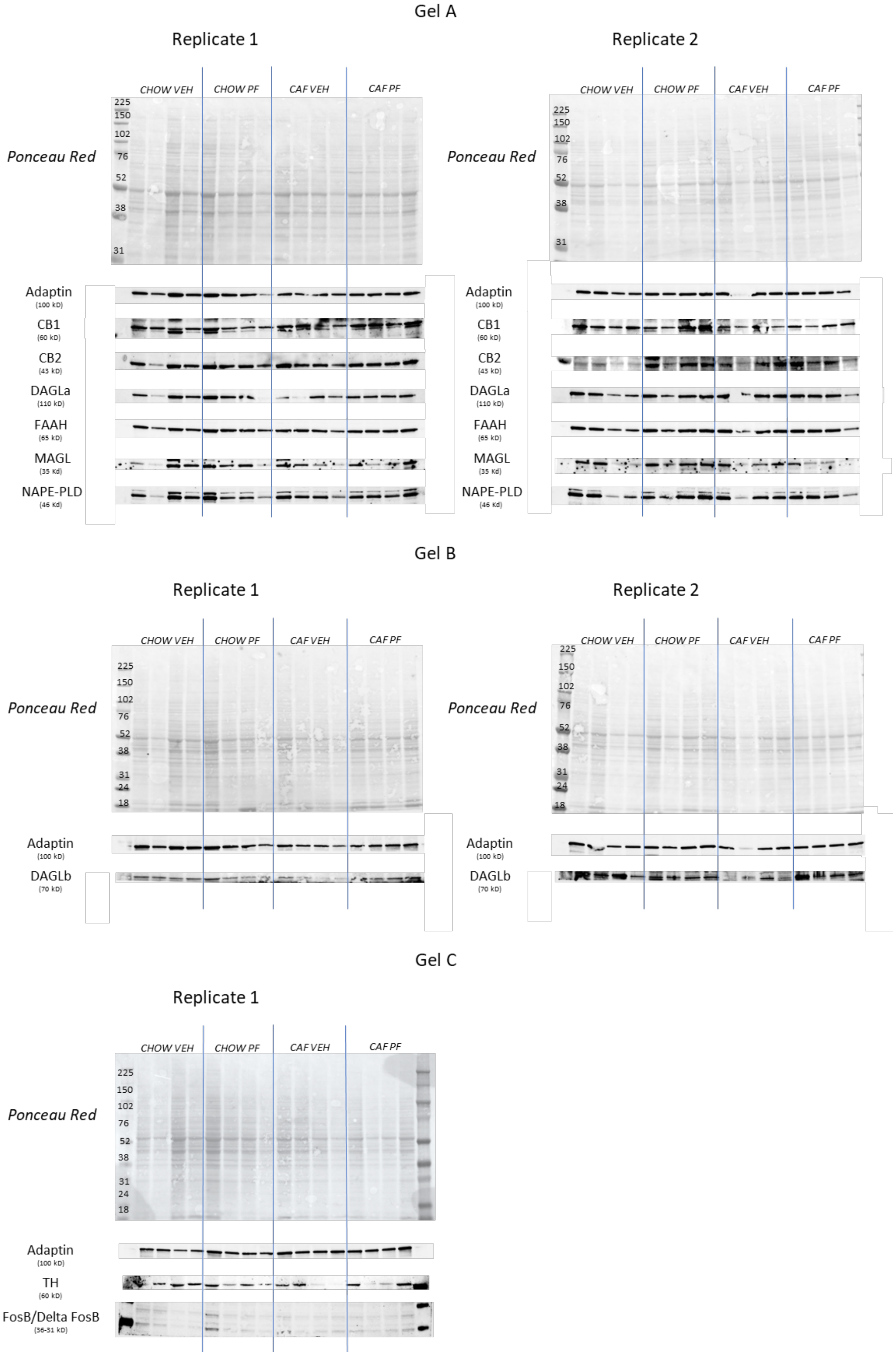

Figure S15. Ponceau Red and complete staining of bands used for Western blot analysis of SN.

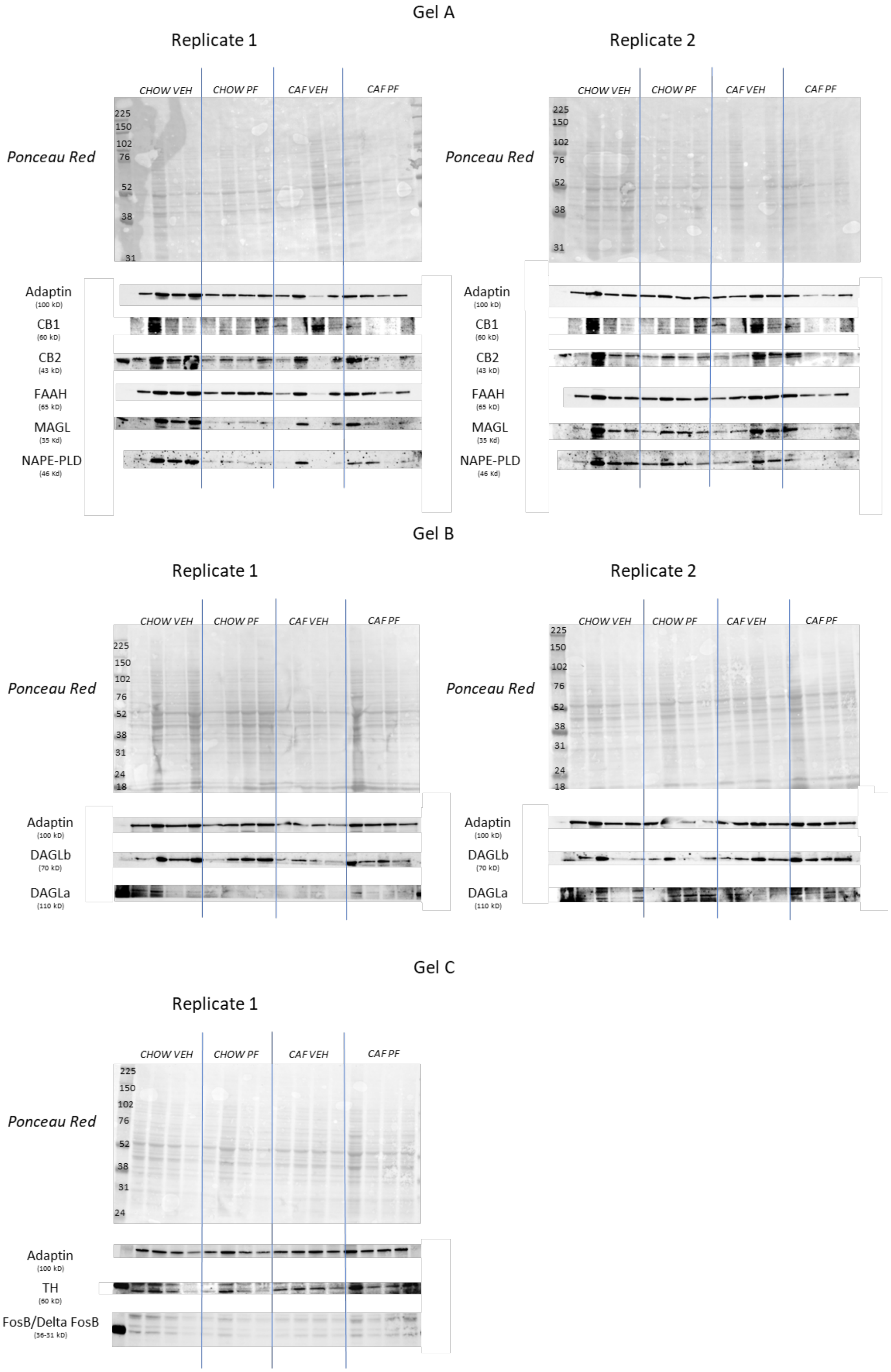

Figure S16. Ponceau Red and complete staining of bands used for Western blot analysis of DLS.

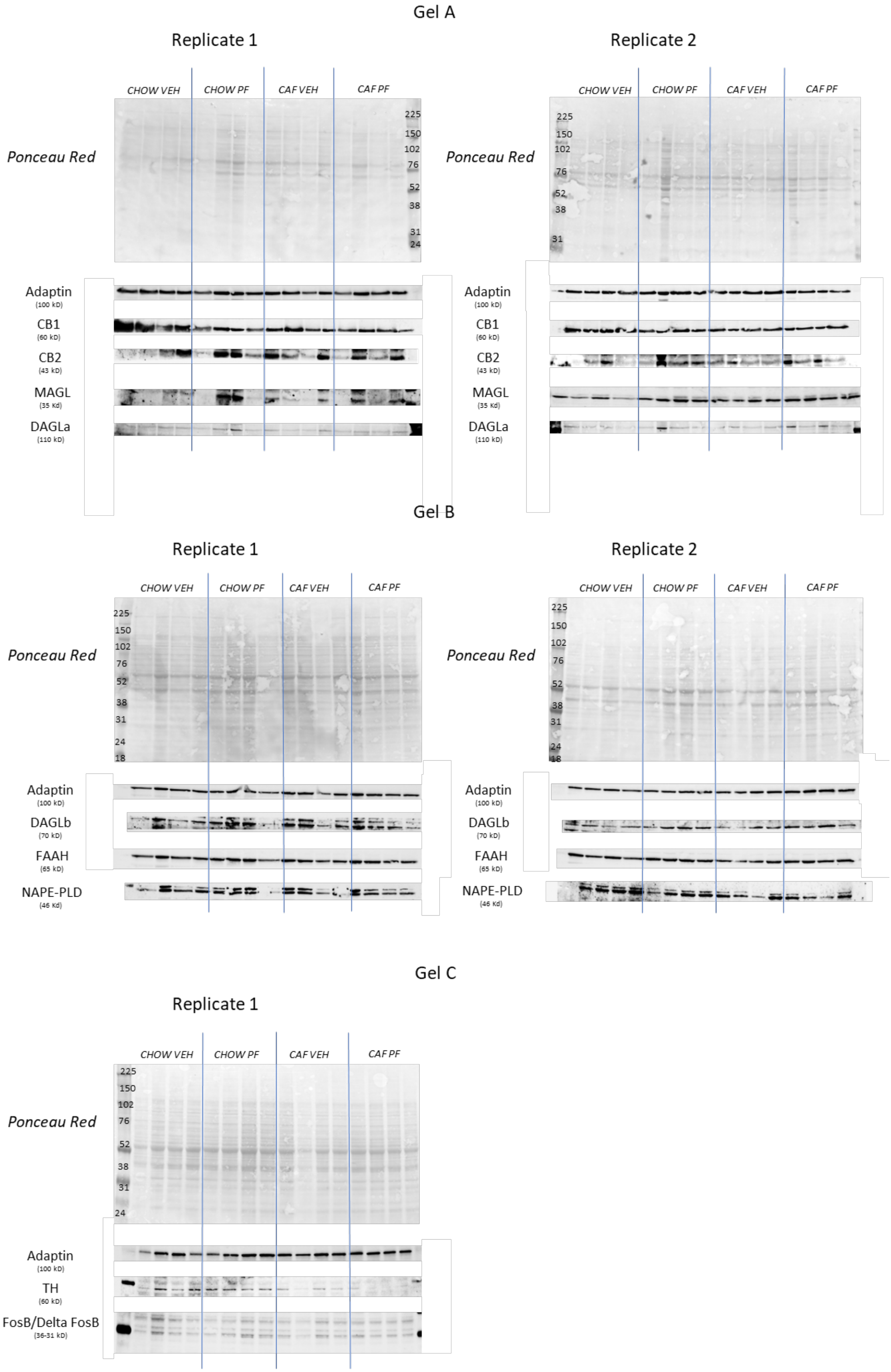

Figure S17. Ponceau Red and complete staining of bands used for Western blot analysis of mPFC.

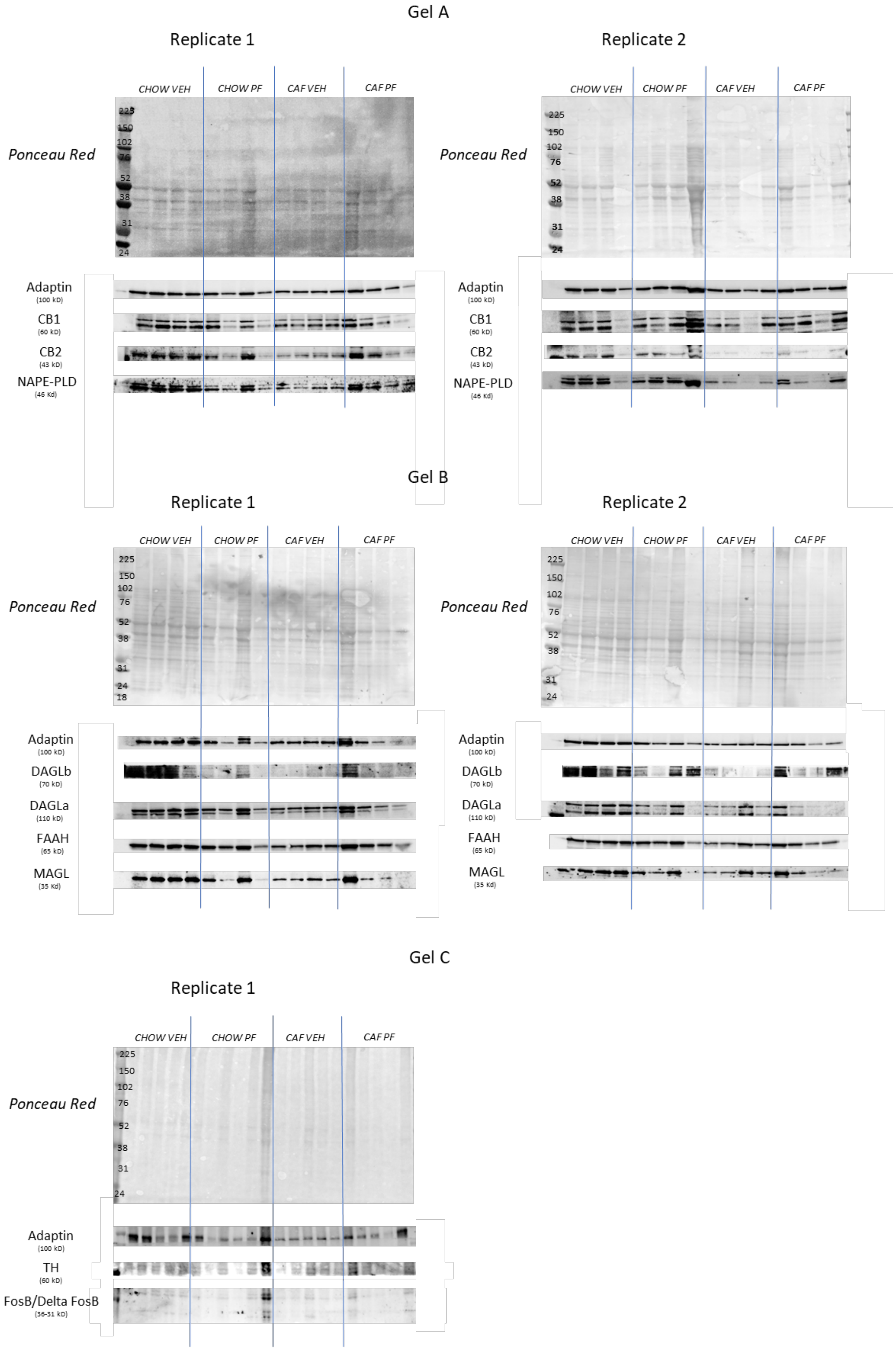

Figure S18. Ponceau Red and complete staining of bands used for Western blot analysis of PFC.

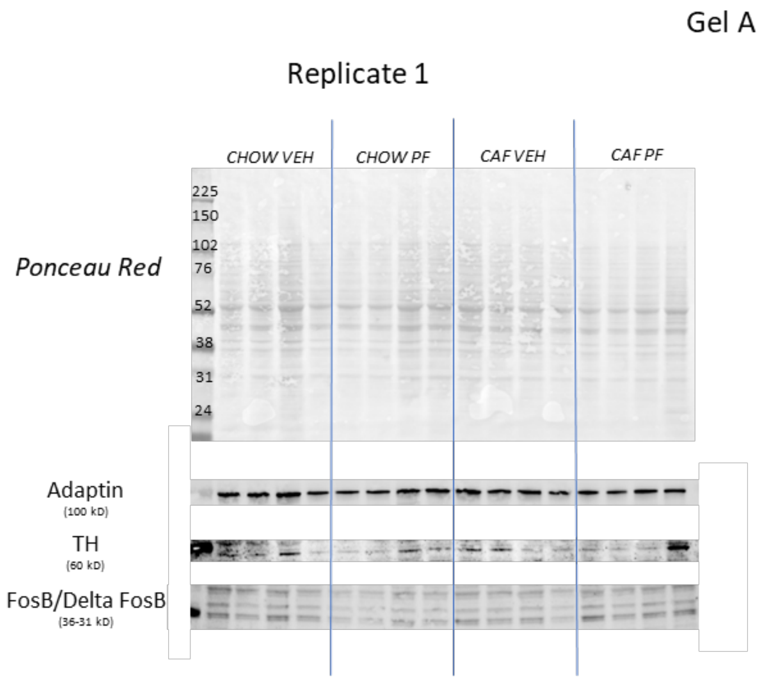

Figure S19. Ponceau Red and complete staining of bands used for Western blot analysis of ACC.

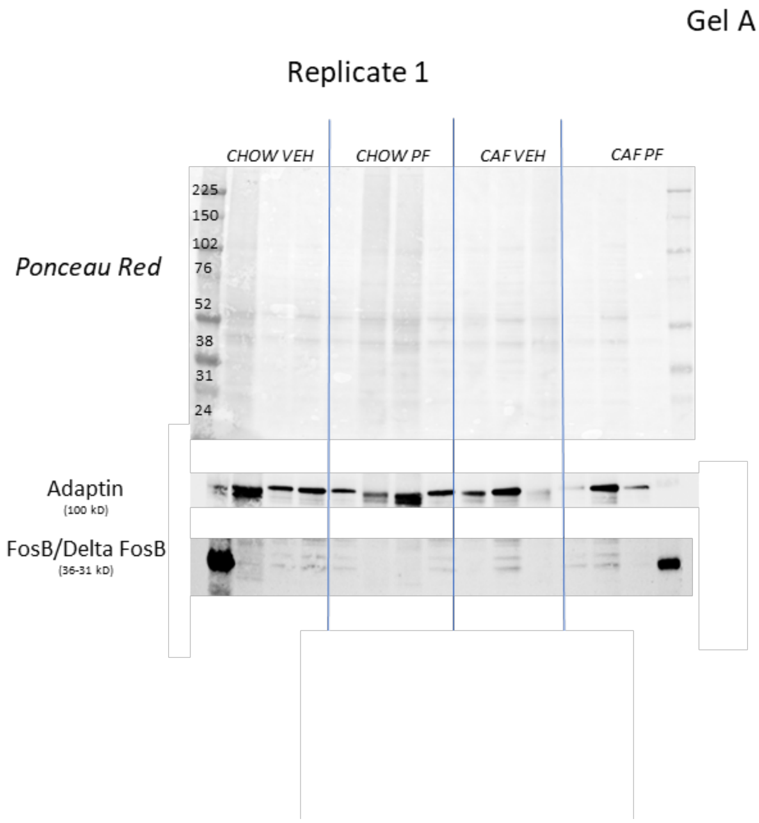

Figure S20. Ponceau Red and complete staining of bands used for Western blot analysis of DR.

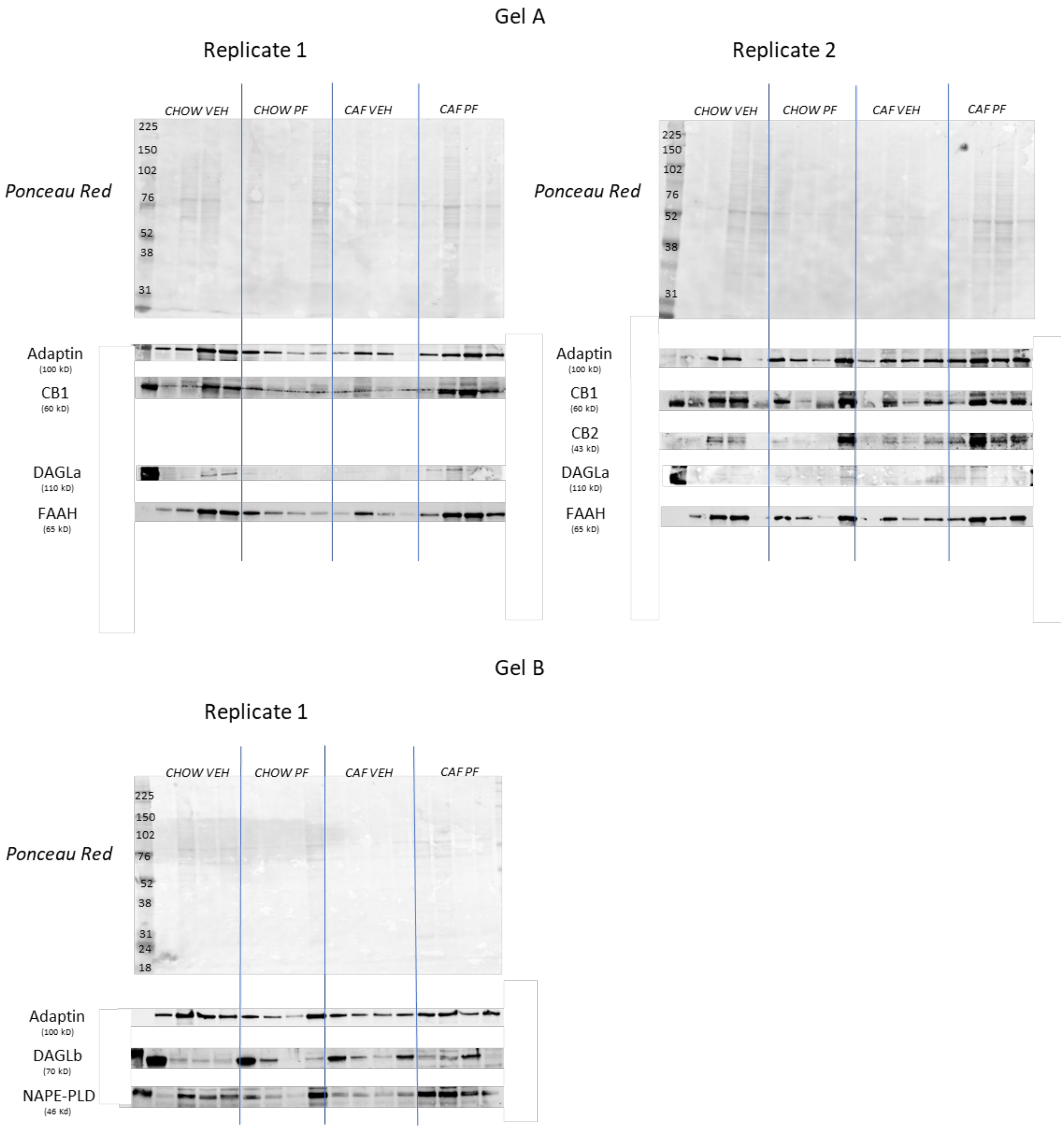

S3.1 Supplementary Biography:

[1] Cassano, T., Gaetani, S., Morgese, M. G., Macheda, T., Laconca, L., Dipasquale, P., Taltavull, J., Shippenberg, T. S., Cuomo, V., & Gobbi, G. (2009). Monoaminergic changes in locus coeruleus and dorsal raphe nucleus following noradrenaline depletion. *Neurochem Res*, *34*(8), 1417-1426. https://doi.org/10.1007/s11064-009-9928-5

[2] Cifani, C., Micioni Di Bonaventura, E., Botticelli, L., Del Bello, F., Giorgioni, G., Pavletić, P., Piergentili, A., Quaglia, W., Bonifazi, A., Schepmann, D., Wünsch, B., Vistoli, G., & Micioni Di Bonaventura, M. V. (2020). Novel Highly Po-

tent and Selective Sigma1 Receptor Antagonists Effectively Block the Binge Eating Episode in Female Rats. *ACS Chem Neurosci*, *11*(19), 3107-3116. https://doi.org/10.1021/acschemneuro.0c00456

[3] Porsolt, R. D., Le Pichon, M., & Jalfre, M. (1977). Depression: a new animal model sensitive to antidepressant treatments. *Nature*, *266*(5604), 730-732. https://doi.org/10.1038/266730a0

[4] Vitale, G., Filaferro, M., Micioni Di Bonaventura, M. V., Ruggieri, V., Cifani, C., Guerrini, R., Simonato, M., & Zucchini, S. (2017). Effects of [Nphe(1), Arg(14), Lys(15)] N/OFQ-NH(2) (UFP-101), a potent NOP receptor antagonist, on molecular, cellular, and behavioral alterations associated with chronic mild stress. *J Psychopharmacol*, *31*(6), 691-703. https://doi.org/10.1177/0269881117691456

[5] Yankelevitch-Yahav, R., Franko, M., Huly, A., & Doron, R. (2015). The forced swim testiss a model of depressive-like behavior. *J Vis Exp*(97). https://doi.org/10.3791/52587
